# Transcriptome analyses of *Ditylenchus destructor* in responses to cold and desiccation stress

**DOI:** 10.1590/1678-4685-GMB-2018-0057

**Published:** 2020-03-23

**Authors:** Juan Ma, Bo Gao, Rongyan Wang, Xiuhua Li, Shulong Chen

**Affiliations:** 1 Institute of Plant Protection, Hebei Academy of Agricultural and Forestry Sciences /IPM centre of Hebei Province/ Key Laboratory of Integrated Pest Management on Crops in Northern Region of North China, Ministry of Agriculture, Baoding, China.

**Keywords:** Potato rot nematode, transcriptome, tolerance, differentially expressed genes

## Abstract

The objective of this study was to identify molecular responses in *Ditylenchus destructor* to cold and desiccation by means of transcriptomes analyses. A total of 102,517 unigenes were obtained, with an average length of 1,076 bp, in which 58,453 (57%) had a functional annotation. A total of 1154 simple sequence repeats (SSRs) distributed over 1078 unigenes were detected. Gene expression profiles in response to cold and desiccation stress and the expression of specific stress-related genes were compared. Gene ontology analysis and pathway-based analysis were used to further investigate the functions of the differentially expressed genes. The reliability of the sequencing data was verified through quantitative real-time PCR analysis of 19 stress-related genes. RNA interference used to further assess the functions of the cold-related unigenes 15628 and 15596 showed that the knockdown of each of these genes led to decreased cold tolerance of *D. destructor*. Hence, this study revealed molecular processes and pathways active in cold- or dessication-treated nematodes. The transcriptome profiles presented in this study provide insight into the transcriptome complexity and will contribute to further understand stress tolerance in *D. destructor.*

## Introduction

The potato rot nematode *Ditylenchus destructor* has become a serious problem to agriculture worldwide, mostly in temperate regions (EPPO, 2018). In addition to potato, *D. destructor* may feed on more than 100 species of plants, and on the mycelia of over 65 species of soil-inhabiting fungi (Sturhan and Brzeski, 1991). China accounts for the highest sweetpotato (*Ipomoea batatas* (L.) Lam.) production in the world (FAOSTAT, 2016) and sweetpotato is the main *D. destructor* host in China. The nematode damages sweetpotato roots, sprout, stem and vine (Qi *et al.*, 2008). As a migratory endoparasite, all stages of *D. destructor* can be found either in the host plant tissues or in the surrounding soil. It can continue to live and reproduce within tuberous roots in storage. Once established, *D. destructor* is difficult to eliminate from the field. Sweetpotato yield losses due to *D. destructor* damage range from 10% to 30%, and up to 100% under heavy infestations (Wang *et al.*, 2018). *Ditylenchus destructor* is particularly widespread in north China (Qi *et al.*, 2008), which features a temperate climate with very cold and dry winters. According to our own observation data in Baoding, Hebei, China from 2015 to 2018, the average soil temperature at 10 cm depth below the soil surface is -1 to -3 °C in January. Despite the harsh conditions, this nematode could be extracted from infected sweetpotatoes which were abandoned in the field after a cold winter and can also be isolated from completely dry sweetpotatoes (data not shown). *Ditylenchus destructor* may have adaptation mechanisms in response to prolonged cold and desiccation pressure.

Molecular mechanisms involved in nematode cold or desiccation tolerance are far from fully understood. Freeze avoidance and freezing tolerance are the main cold tolerance strategies by which nematodes can survive cold (Wharton, 2011). The Antarctic nematode *Panagrolaimus davidi* expresses the recrystallization-inhibition protein (RIP) that inhibits recrystallization but has little thermal hysteresis activity (Wharton *et al.*, 2005; Adhikari and Adams, 2011), this protein is relatively heat stable and present at low concentrations. The Antarctic soil nematode *Plectus murrayi* survives low temperatures by either freezing tolerance or cryoprotective dehydration (Wharton and Raymond, 2015). Ohta *et al.* (2014) reported that the nervous system and intestine regulate the cold tolerance of *Caenorhabditis elegans*, where temperature is sensed by a thermo-sensing neuron that induces insulin secretion. Trehalose plays an important role in nematode cold tolerance strategies; it depresses the supercooling point of a solution and assists cold tolerance by freeze avoidance (Ring and Danks, 1998). The free-living nematode *Aphelenchus avenae* accumulated large amounts of the disaccharide trehalose, late embryogenesis abundant (LEA) protein and anhydrin protein following exposure to a moderate reduction in relative humidity (Goyal *et al.*, 2005). Trehalose metabolism genes were reported to play important role in hypertonic osmotic pressure in the rice white tip nematode *Aphelenchoides besseyi* (Chen *et al.*, 2017). Antifreeze proteins (AFPs) can prevent ice nucleation by producing a thermal hysteresis, thus play an important role in freeze tolerance in nematodes (Adhikari *et al.*, 2010; Ali and Wharton, 2016). Heat shock proteins (Hsps) are induced by various stressors in many nematodes (Adhikari *et al.*, 2009; Wharton, 2011); for instance, small HSPs are essential for desiccation tolerance of *C. elegans* (Erkut *et al.*, 2013). LEA proteins are widespread to protect cells against water loss and they are important for anhydrobiosis in both animals and plants (Wharton, 2011). Silencing the expression of a LEA gene in *C. elegans* led to reduced survival of desiccation and heat stress (Gal *et al.*, 2004); LEA protein 3 production was elevated in infective juveniles of the entomopathogenic nematode *Steinernema feltiae* during desiccation stress (Gal *et al.*, 2001). Expression of DAF-16 target genes in *C. elegans* promotes tolerance to low temperatures (Savory *et al.*, 2011).

Transcriptome analyses of other species have contributed to the understanding of the molecular mechanisms involved in cold or desiccation stress responses (Adhikari *et al.*, 2010; Thorne *et al.*, 2014; Yaari *et al.*, 2016). However, cold and desiccation abilities vary between different nematode species and involve several mechanisms. vilponis *et al.* (2011) examined the cold tolerance of *D. destructor* in various aqueous environments as well as in potato tissues. After 24 h exposure at -5 °C, *D. destructor* mortality in water and gravel was significantly higher than in potato and M9 buffer. Nematode infested potato tissues were then exposed to -5, -8, -18 and -30 °C for 24 h, and the result showed that the younger juveniles are significantly more tolerant than adults and fourth-stage juveniles. Peng *et al.* (2013) generated 9800 ESTs from a *D. destructor* mixed-stage library Twenty two sequences were similar to published nematode effectors, and 391 secretome members were identified from these ESTs. Resistance to cold and desiccation stress in the field is important to *D. destructor* survival, although limited information on the genes acting in these processes is available. Here we report a transcriptome analysis of gene expression patterns during the adaptation to cold and desiccation stress.

## Materials and Methods

### Nematode culture and sample preparation

*Ditylenchus destructor* was originally isolated from diseased sweetpotato in Yixian, Hebei, China (N39.35, E115.48) and has been maintained on sweetpotato (25 °C, 70-80% relative humidity) for more than one year. The average annual precipitation in Yixian is 538 mm in the last decade and is concentrated mostly in summer. Soil texture is mainly sandy loam. Mixed stages of *D. destructor* were collected from infected sweetpotato by the modified Baermann funnel method 30 days after inoculation (Wang *et al.*, 2016). The extracted nematodes were cleaned with sterile distilled water. Nematodes maintained under the same conditions for the same time were used for all the experiments.

### RNA extraction and RNA-seq library construction

Our previous experiments indicated that the mortalities of *D. destructor* significantly increase after 35 days exposure to 10% glycerol solution or 5 days exposure to 20%. To avoid death, four nematode treatments were set up. The cold stress group nematodes were exposed to water at -1 °C for 35 d (D_1W) and -3 °C for 35 d (D_2W). The desiccation stress group nematodes were exposed to 10% glycerol for 35 days (D_1G) and 20% glycerol for five days (D_2G). The control group consisted of non-treated nematodes from the same batch as the desiccated and cold treated counterparts, kept in distilled water at 25 °C for 35 days (CK_6). For each sample, millions of nematodes (~200 mg) were pooled and immediately snap frozen with liquid nitrogen and then stored at -80 °C until used for RNA extraction.

Total RNA was extracted individually from each sample using Trizol reagent (Invitrogen, Life Technologies, USA). The RNA was subsequently treated with RNase free DNase I (Invitrogen, USA) to remove DNA contamination according to the manufacturer’s protocol. RNA integrity was assessed via an Agilent 2100 Bioanalyzer (Agilent, Santa Clara, CA, USA). RNA samples with high purity and high integrity (RNA Integrity Number ≥ 9.0) were used for cDNA library construction. The experimental and control groups consisted of three biologically independent RNA preparations each. RNA from the three replicates of each sample was combined for RNA-seq. The RNA-seq libraries were constructed following the manufacturer’s instructions (Illumina Inc, San Diego, CA, USA). In brief, poly(A) mRNA was purified from 6 μg total RNA using oligo(dT) magnetic beads, followed by fragmentation of the mRNA with fragmentation buffer. The cleaved RNA fragments served as template for first strand cDNA synthesis using reverse transcriptase and random hexamers. Second strand cDNA synthesis was done using DNA polymerase I and RNaseH. The double stranded cDNA was end-repaired and ligated to adaptors after adding a single “A” at the 3’ end. The fragments with a size suitable for sequencing were isolated from the gels and enriched by PCR amplification to construct the final cDNA library. Finally, the cDNA library was sequenced on an Illumina HiSeq^TM^4000 platform (BGI-tech, Shenzhen, China). Raw sequencing data were submitted to NCBI Sequence Read Archive under accession number SRR5234500-SRR5234504.

### *De novo* transcriptome assembly and functional annotation

The raw reads were cleaned by removing the adaptor sequences, poly-N and low quality sequences. The quality reads were *de novo* assembled into transcripts using Trinity v2.0.6, with an optimized K-mer length of 25 and all other parameters set to default (Haas *et al.*, 2013). TGICL v2.0.6 was used to cluster transcripts to unigenes (Pertea *et al.*, 2003). The unigenes were annotated by aligning with the deposited ones in databases, such as NCBI non-redundant protein (Nr), Gene Ontology (GO), Swiss-Prot protein database, Kyoto Encyclopedia of Genes and Genomes (KEGG) pathway database, Cluster of Orthologous Groups (COG) database, and Interpro using BLASTX (E-value ≤ 1e^-5^). Sequence orientations and the protein coding region sequence (CDS) of unigenes were determined according to the best hit in the database. ESTScan (Iseli *et al.*, 1999) was used to predict the sequence direction and CDS when unigenes were unaligned to any of the databases. With Nr annotation, the Blast2GO program (v2.5.0) with default parameters was used to obtain GO annotation of the unigenes (Conesa *et al.*, 2005). After GO annotation for every unigene, WEGO software (Ye *et al.*, 2006) was used for GO functional classification and graphical representation of GO terms (Zhang *et al.*, 2017). InterProScan5 was used for InterPro annotation (Quevillon *et al.*, 2005). Blast results information was used to extract CDS from unigene sequences and translate them into peptide sequences. To identify potential novel genes, non-annotated unigenes were predicted by ESTScan and translated into peptide sequences.

### Screening for SSRs and SNPs

Potential simple sequence repeats (SSRs) marks were searched by the software MicroSatellite (http://pgrc.ipk-gatersleben.de/misa/). Six types of SSRs with repeats of one to six nucleotides in length were investigated. The parameters were set to identify mono-, di-, tri-, tetra-, penta-, and hexa-nucleotide motifs with a minimum of twelve, six, five, five, four, and four repeats, respectively. The maximal distance was 100 nucleotides interrupting two adjacent distinct SSRs in a compound microsatellite. After assembly, all clean reads were mapped to unigenes using HISAT (Kim *et al.*, 2015). The assembled contigs were scanned for single nucleotide polymorphisms (SNPs) with GATK (McKenna *et al.*, 2010). The final SNPs were obtained after filtering out unreliable sites.

### Differentially expressed genes

Clean reads were mapped to unigenes using Bowtie2 (Langmead and Salzberg, 2012), and gene expression levels were calculated by RSEM (Li and Dewey, 2011). Reads per kilo base of transcript sequence per million mapped reads (RPKM) for each unigene were calculated for gene expression profiles (Mortazavi *et al.*, 2008). The False Discovery Rate (FDR) was calculated by the Benjamini and Hochberg’s algorithm (Benjamini and Yekutieli, 2001). Differentially expressed genes between two groups were identified with defaulted filtering criteria of FDR ≤ 0.001 and the absolute value of fold changes (log_2_Ratio) ≥ 2 (Wang *et al.*, 2010). Pairwise and multi-condition analyses were used to detect differentially expressed genes (DEGs) between two samples and among the four different treated nematode samples*.* For the functional and pathway enrichment analysis, the DEGs were then mapped to GO terms and the KEGG database. Significantly enriched GO and KEGG terms were determined by corrected *P* ≤ 0.05. Hierarchical cluster analysis for the DEGs was performed with the pheatmap R package v1.0.8 (http://CRAN.R-project.org/package=pheatmap).

### Quantitative Real-Time PCR validation

Nineteen differentially expressed genes involved in the cold and dessication tolerance of *D. destructor* were randomly selected for quantitative real-time PCR (qPCR) analysis. Specific primers of selected genes were designed by Primer Premier 5.0 (Table S1). Total RNA of the control and treated nematodes was extracted separately as described above and reverse transcribed to first-strand cDNA using reverse transcriptase (TaKaRa). Real-time PCR assays were performed on an ABI Step-One Plus Real Time PCR System (Applied Biosystems) using the KAPA SYBR fast qPCR kit (Kapa Biosystems, Woburn, MA, USA) according to the manufacturer’s protocol in a total volume of 20 μL, containing 1 μL of cDNA, 0.4 μL of 10 μM of each primer, and 10 μL 2SYBR qPCR mixture. The PCR was performed at 95 °C for 5 min, followed by 40 cycles of 95 °C for 15 s, 60 °C for 30 s. β-actin was used as reference gene. qPCR was carried out with three biological replicates and three technical replicates. Melting curve analysis was conducted from 60 to 95 °C. Each of the two primer pairs for the tested genes and endogenous references produced a single peak in the melting curve analyses. Relative gene expression was calculated by the 2^-DDCt^ method (Pfaffl, 2001). The significant difference of 2^-DDCt^ among the different treatments of *D. destructor* was analyzed using the Student’s *t*-test with SPSS Statistics 16.0.

### RNA interference analysis

Two highly expressed genes (unigene 15596 and 15628) revealed in the RNA-seq experiments of cold treated nematodes were selected for RNAi experiments. To check whether the silencing of these genes influenced the nematode’s cold tolerance, a soaking experiment with double-stranded RNA (dsRNA) was set up. The dsRNAs were generated for the selected genes following the instructions of the T7 RiboMAX Express RNAi System (Promega, Madison, Wisconsin, USA) using the primers listed in Table S1. dsRNA of the gene *gfp* was used as non-native negative control. RNAi soaking was performed by a method modified from Wang *et al.* (2017). *Ditylenchus destructor* (~15 mg) were soaked in 200 μL of target dsRNAs solution (unigene 15596, unigene 15628 and *gfp*) at a final concentration of 1.5 μg/μL, and then incubated in a rotator shaker (100 rpm) at 25 °C for 12 h. As control treatments, nematodes were soaked in ddH_2_O. After soaking, the viability of the nematodes from the four samples was evaluated by counting the number of living and dead nematodes in each sample. This experiment was repeated thrice. Afterwards, the nematodes from each above treatment were washed. The mRNA expression level of the target genes and adaptability of the nematodes to cold stress were evaluated. qPCR was used to evaluate the mRNA expression levels of unigene 15596 and 15628 after RNAi. The capacity of *D. destructor* to adapt to cold stress after silencing the unigenes 15596 and 15628 was evaluated according to a previously described method (Ma *et al.*, 2013). Briefly, 1000 nematodes suspended in 1 mL of 5% glycerol were placed in individual wells of a 24-well plate. The nematodes in the plates were exposed to -8 °C for 4 d. After cold treatments, nematodes were transferred to Petri dishes (60 mm diameter) containing 9 mL of distilled water and incubated at 25 °C for 24 h to allow recovery of the nematodes. Nematode survival was assessed by microscopic observation, and nematodes were considered dead when showing no response after probing with a needle. Untreated nematodes were used as controls in each of the experiments. Six replicates were run for each sample.

### Statistical analysis

Effects of cold or desiccating conditions on nematode mortality were analyzed with SPSS Statistics 16.0. Data from repeated bioassays that were not significantly different were pooled for analysis. Statistical analyses were performed using one-way or two-way ANOVA, followed by Tukey’s multiple means comparison procedure. Percentage data were arcsine transformed before analysis. A *P* value < 0.05 was considered statistically significant.

## Results

### Transcriptome sequencing and assembly

Each run yielded about 4.5 gigabases (Gb) clean paired end reads, and a total of 22.48 Gb clean data were obtained with cycle Q20 (base quality greater than 20) > 96% (Table 1). The GC content of the transcriptomes spanned from 42.31% to 44.05%. Quality reads from each library were *de novo* assembled separately into 102,517 unigenes, with an average length of 1,076 bp and the N50 statistical value was 1,870 bp. The unigenes total length was 110,389,848 bp.

**Table 1:** Statistics of reads in the *Ditylenchus destructor* transcriptomes.

	CK_6	D_1G	D_2G	D_1W	D_2W
Clean reads (Mb)	29.99	29.92	29.85	30.05	29.97
Total clean bases (Gb)	4.50	4.49	4.48	4.51	4.50
Clean reads ratio (%)	91.82	91.60	91.38	92.01	91.75
Clean reads Q20 (%)	96.22	96.42	96.59	96.31	96.44
Number of transcripts	88 480	86 694	90 581	76 250	76 643
Number of unigenes	60 018	57 932	62 469	51 729	51 466
Unigenes GC contents (%)	42.58	42.59	42.31	43.43	44.05

### Unigene annotation and classification

Unigene sequences were aligned by BLASTX (E-value ≤ 1e^-5^) to Nr, Nt, Swiss-Prot, GO, COG, KEGG and Interpro databases. Protein function information was predicted from annotation of the most similar protein in these databases. A total of 58,453 unigenes were annotated with known biological functions, comprising 57% of all the unigenes in the transcriptome libraries. In total, 55,320 (54%) unigenes were annotated against Nr database. For the best-matched species distribution, 22.3% of the distinct sequences mapped to the sequences of *Ascaris suum*, 12.3% aligned to the sequences of *Ancylostoma ceylanicum*, 10.8% to *Loa loa,* and 8.3% mapped to *Haemonchus contortus.* The searches revealed that 12,227 (11.9%), 46,874 (45.7%), 23,796 (23.2%), 44,135 (43.1%), 28,349 (27.7%) and 45,757 (44.6%) of the unigenes had similarity in the Nt, Swiss-Prot, COG, KEGG, GO and Interpro databases, respectively. After functional annotation, 55,476 CDS were detected. CDSs of unigenes that had no hit in blast searches were predicted by ESTScan, and 14,536 unigenes obtained significant blast hits.

All the unigenes were functionally characterized into GO categories based on their similarities to known proteins in Nr database. Sequences were classified into 58 subcategories belonging to three main GO categories, including biological process (24 subcategories), cellular component (17 subcategories), and molecular function (17 subcategories) (Figure S1A). Within the biological process category, single-organism process and cellular process were predominant. In the category of cellular components, ‘cell part’ and ‘cell’ were dominant over the other subgroups. Binding and catalytic activity were the most representative subcategories in the molecular function category. Additionally, pathways were predicted from the KEGG database, and in total, 44,135 unigenes were grouped into 42 pathways (Figure S1B). The most dominant clusters were related to signal transduction.

### SSR and SNP detection

**Figure 1:**
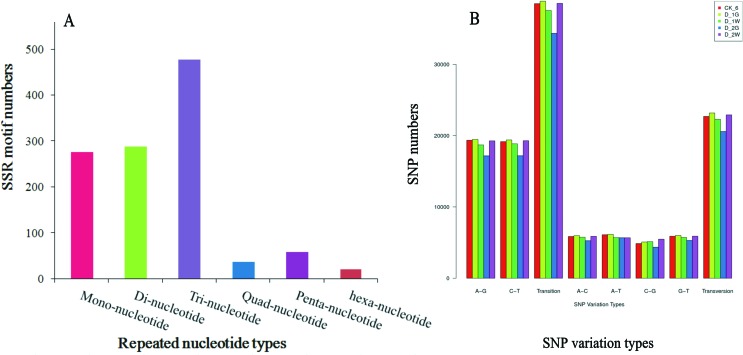
Simple sequence repeats (SSRs) size distributions (A) and Single nucleotide polymorphisms (SNPs) variant type distributions (B). X axis represents the type of SSRs/ SNP, Y axis represents the number of SSRs/ SNPs.

Simple sequence repeats have commonly been used in genetic diversity, molecular assisted selection, population structure, and genetic mapping studies. SSRs in genes that occur in the protein-coding regions of annotated unigenes could be used to analyze the attributes of functional genes in association with their phenotypes (Zalapa *et al.*, 2012). The transcriptome data was used to analyze the SSRs, because no information is available for SSRs in *D. destructor*. A total of 1154 SSRs distributed over 1078 unigenes were detected. Tri-nucleotides were the most frequent motif types, followed by di-nucleotide and mono-nucleotide. Among the microsatellites detected, the dominant classes of repeating sequences in the unigenes were AT/AT (154) representing 53.5% of the di-nucleotide SSRs, followed by AAT/ATT (123) representing 25.8% and AAG/CTT (97) representing 20.3% of the tri-nucleotide SSRs (Figure 1A). A complete list of SSRs is provided in Table S2.

Due to their wide distributions and abundant polymorphisms, SNPs are useful markers for investigating population genetic structures, the mapping important traits, and for whole-genome association studies (Luo *et al.*, 2016). Functional SNP markers could provide genotype information as they occur in coding regions that may cause a loss or change of protein function (Ellegren, 2008). Each transcriptome generated nearly 60,000 SNPs (Figure 1B). Transitions were more abundant than transversions. The numbers of the transition type A/G and C/T were equally abundant, and the numbers of the transversion types A/T, A/C, and G/T were in the similar level. The statistics of the SNPs validated in all samples are in Table S3.

### Changes in gene expression profile among the different nematode samples

**Figure 2:**
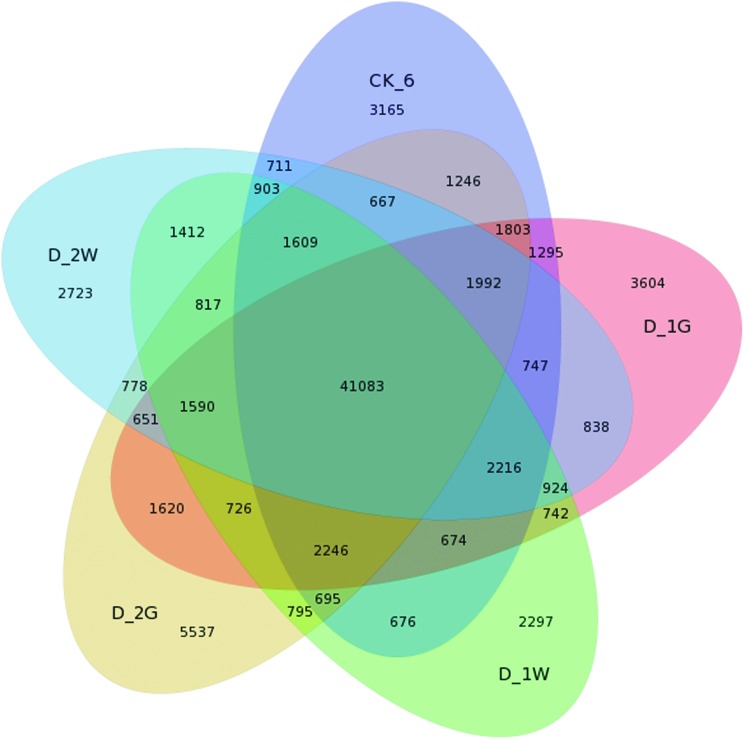
Venn diagram illustrating common and specifically unigenes in different treatments of Ditylenchus destructor.

Genes that were highly expressed in both cold-treated samples were considered as cold related genes, and genes that were highly expressed in both desiccation-treated samples were considered as desiccation-related (FDR ≤ 0.001 and log_2_Ratio ≥ 2). A Venn diagram displays the number of unique and shared DEGs amongst the five nematode samples, 41,083 unigenes were shared across all five libraries (Figure 2). The analysis of differentially expressed genes in response to cold revealed 4743 common unigenes in D_1W, and D_2W, 9303 were specific expressed in D_1W and 9,733 unigenes were specific expressed in D_2W. 43% of these shared unigenes have no annotation. Compared with the control, nematodes in cold conditions had notably higher expression levels for unigenes related to mucin 5B, serine/threonine protein phosphatase, antifreeze protein, histone acetyl transferase, RAB11 family interacting protein, neuropeptide-like protein 29 (Nlp-29), udp-glucuronosyltransferase and trichohyalin-like (Table S4). Compared with the control nematodes (CK_6), 10,695 and 12,514 unigenes were specifically observed in D_1G and D_2G respectively. Furthermore, 4,587 unigenes were shared across the two desiccation treated samples (Figure 2). For 49% of these shared unigenes no annotation was found. The highly expressed annotated unigenes included those encoding mucin-5B, GMP synthase, dihydrodiol dehydrogenase, CBR-NURF-1 protein, ubiquitin C and lipid phosphate phosphohydrolase (Table S4).

**Figure 3:**
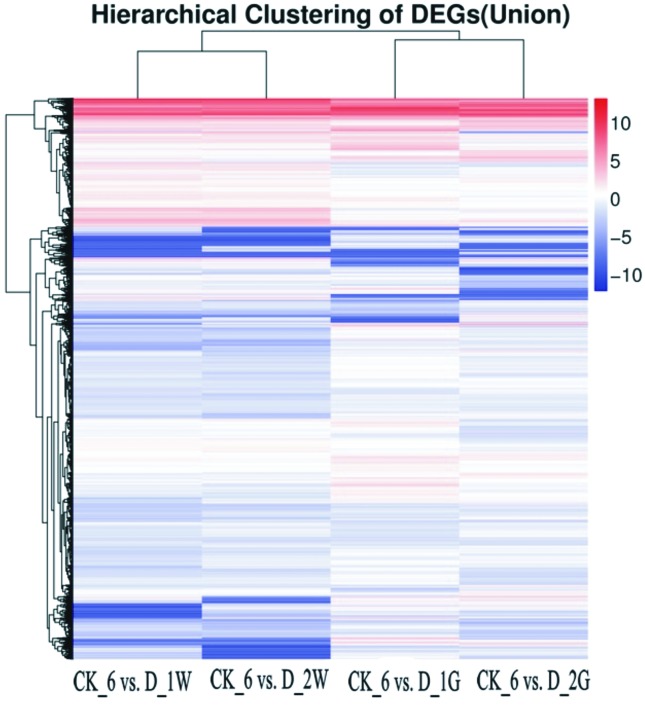
Heatmap of hierarchical clustering of differentially expressed genes (DEGs). X axis represents each comparing samples. Y axis represents DEGs. Coloring indicates fold change.

Differentially expressed unigenes between pairwise combinations of different nematode samples were identified (Figure S2). In nematodes exposed to cold or desiccation conditions, a number of genes were induced during the nematode acclimation process, and expression of many genes was shut down during the adaption process (Table 2). Compared to the control, nematodes in the cold conditions showed a significantly higher number of down-regulated DEGs than the nematodes in dry conditions, suggesting that more genes were affected by the cold stress. A hierarchical cluster analysis was used to determine the profiles of the DEGs from pairwise comparisons among the control nematodes and the treated nematodes (Figure 3). Unigenes with similar expression patterns were grouped in the same cluster. The analysis showed that the unigenes’ expression pattern in the D_1W treatment was similar to that of the D_2W, and the unigenes’ expression pattern of D_1G was similar to that of the D_2G.

**Table 2:** Number of differentially expressed genes (DEGs) in pairwise comparisons between different nematode samples.

Samples	Up-regulated	Down-regulated
CK_6 *vs.*D_1G	5907	3911
CK_6 *vs.*D_2G	4815	5124
CK_6 *vs.*D_1W	5125	8556
CK_6 *vs.*D_2W	5192	7846

### Functional annotation of differentially expressed unigenes

We compared the differences occurring between the control and all treated nematodes (Figure S3). With regard to DEGs between the control nematodes and the cold treated nematodes (D_1W and D_2W), the DEGs were significantly enriched (*P* < 0.05) in terms such as sugar-phosphatase activity, cation channel activity, and voltage-gated channel activity. Specifically, GO enrichment showed that the terms of pre-mRNA 3’-splice site binding, voltage-gated ion channel activity, and voltage-gated channel activity were only significantly enriched in cold conditions. Phosphofructokinase activity was significantly enriched in desiccation conditions. Furthermore, GO terms serine-type carboxypeptidase activity, serine-type exopeptidase activity, and alpha-1,3-mannosylglycoprotein 2-beta-N-acetylglucosaminyltransferase activity were significantly up-regulated in the cold treated nematodes (D_1W and D_2W). The GO terms stearoyl-CoA 9-desaturase activity, 6-phosphofructo-2-kinase activity, and fructose-2,6-bisphosphate 2-phosphatase activity showed significant up-regulation in D_1G, and aryl sulfotransferase activity, structural constituent of ribosome, and structural molecule activity were significantly enriched in up-regulated genes of D_2G (Table S5).

All DEGs were mapped to the KEGG database and compared with the whole transcriptome data. Notably, some pathways were only influenced under certain stresses. For instance 9,656 differentially regulated unigenes with KEGG annotation were identified between the CK_6 and D_1W samples, with specific significantly enrichment of genes (*P* ≤ 0.05) observed for pathways of nitrogen metabolism, seleno-compound metabolism, and PI3K-Akt signaling pathway. Furthermore, 9,471 DEGs were found when comparing CK_6 and D_2W, with specific significantly enrichment of hypertrophic cardiomyopathy, other glycan degradation, biotin metabolism, and the serotonergic synapse pathways. In D_1W and D_2W, the pathways of retinol metabolism, spliceosome, and pentose and glucuronate interconversions were significantly up-regulated; neuroactive ligand-receptor interaction, calcium signaling pathway, fructose and mannose metabolism, and lipoic acid metabolism were significantly down-regulated (Table S6). Another 6,878 differentially regulated unigenes were identified between the CK_6 and D_1G libraries. Among these, genes associated with glycine-serine and threonine metabolism, biosynthesis of unsaturated fatty acids, arachidonic acid metabolism, linoleic acid metabolism, carbon metabolism, and fatty acid metabolism were functionally specific significantly enriched. A further 7,169 differentially regulated unigenes were identified between the CK_6 and D_2G libraries, with specific significantly enrichment of unigenes for pathways involved in 2-oxocarboxylic acid metabolism, degradation of aromatic compounds, antigen processing and presentation, lysine degradation, fat digestion and absorption, and pyrimidine metabolism. Pathways of chemical carcinogenesis, metabolism of xenobiotics by cytochrome P450, steroid hormone biosynthesis, ascorbate and aldarate metabolism, ether lipid metabolism and PPAR signaling pathway were enriched among the up-regulated genes of D_1G and D_2G; in contrast, long-term potentiation was significantly down-regulated in both D_1G and D_2G (Table S6). The up-regulated pathways of pentose and glucuronate interconversions, retinol metabolism, were commonly shared by all the four treated samples. These results indicate that DEGs involved in these pathways may play an important role in the adaption of harsh environmental conditions.

### Validation of DEGs data by Quantitative RT-PCR

**Figure 4:**
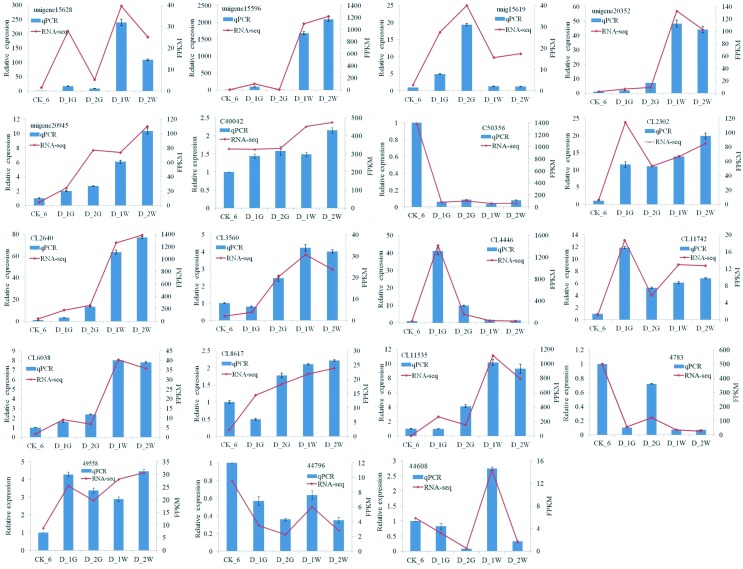
RNA-seq and RT-qPCR analyses of expression levels of tolerance related genes in different nematode samples. X-axis indicates nematode samples, and y-axis indicates gene expression levels by qPCR (left) and FPKM (right). Histograms indicate the relative expression value obtained by qPCR; the solid lines in all plots indicate the FPKM values obtained by RNA-seq.

No amplification was found in no-template controls, and single gene specific melting curves confirmed the specificity of the qRT-PCR assays. Fold changes from qPCR assays were compared with the RNA-seq expression profiles (Figure 4, Table S4). In general, all unigenes showed similar expression patterns with the RNA-seq data, adding confidence to the RNA-seq data analysis.

### The effect of RNAi

**Figure 5:**
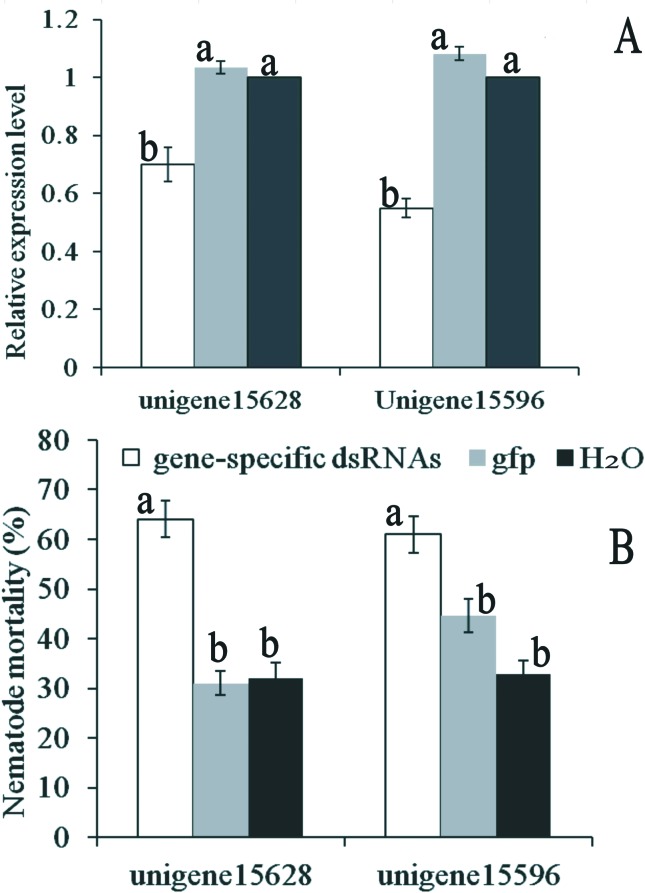
Effect of RNAi on cold tolerance of Ditylenchus destructor. (A) Gene expression analysis by qPCR of unigene 15628 and 15596 after RNAi. Nematodes were incubated in unigene 15628 and 15596 dsRNA for 24 h. Control nematodes were incubated in ddH2O without dsRNA. (B) Mean mortality (± SE) of control and dsRNA-treated nematodes after exposure to -8 °C for 4 days. Silencing unigene 15628 and 15596 promoted a significant increase in mortality (64% ± 3.6%, 61% ± 3.7%) when compared to control nematodes (32% ± 2.9%, 33% ± 2.8%). Bars with the same letter(s) do not differ significantly according to Tukey’s test (P > 0.05).

Compared with the control treatments, the relative expression levels of the unigenes 15628 and 15596 were significantly reduced in the dsRNA-treated nematodes (*F* = 30.78; df = 2, 6; *P* < 0.01; *F* = 129.59; df = 2, 6; *P* < 0.01) (Figure 5A), with an average reduction of 30% for unigene 15628 and 45% for unigene 15596 in the dsRNA soaked nematodes compared to the control group. dsRNA had no effect on the survival rate of *D. destructor*, and nematode survival among different treatments did not statistically differ and remained above 95% before exposure to -8 °C. The effect of silencing the unigenes 15628 and 15596 on cold tolerance was studied (Figure 5B). Nematodes were transferred into 5% glycerol before being exposed to -8 °C. Glycerol was used as plasma membrane-permeant cryoprotectant to inhibit intracellular ice formation (Storey *et al.*, 1998). The unigenes 15628 and 15596 dsRNA-treated nematodes displayed significantly higher mortality compared to the *gfp* dsRNA-treated and none-treated nematodes after subsequent exposure to -8 °C (*F* = 37.57; df = 2, 15; *P* < 0.01; *F* = 18.36; df = 2, 15; *P* < 0.01). Knockdown of each gene led to decreased cold tolerance of *D. destructor*.

## Discussion

The aim of the present study was to characterize and study the genes related to cold and dessication tolerance of *D. destructor.* Differential gene expression levels across control and the treated nematode samples were analyzed. In response to the stress, the nematodes activated metabolic pathways shortly after exposure to different stresses. Not surprisingly, some reported stress-related genes were in some way involved in the stress response, such as genes encoding the LEA and HSP proteins. LEA proteins play an important role during desiccation in nematodes (Adhikari *et al.*, 2010; Erkut *et al.*, 2013). We recorded 14 unigenes related to LEAs in *D. destructor* using BLASTX similarity searches of our transcriptome sequences against the Nr databases, with nine LEA related genes being up-regulated during desiccation and also cold stress of *D. destructor* (Table S4)*.* The HSP families contain various genes that could be either induced by stress treatment or constitutively expressed in nematodes (Yaari *et al.*, 2016). Our results showed that the small HSP21-like and small HSP12.6 proteins were highly expressed following cold stress in *D. destructor*. HSP20 and HSP70-A were highly up-regulated during exposure to desiccation, whereas other heat shock proteins in *D. destructor* were constitutively expressed or slightly up or down-regulated. The induction or inhibition of various heat shock proteins might be necessary for *D. destructor* to adapt to cold or desiccation conditions.

The TPS and aquaporin proteins were reported to enhance desiccation resistance in many nematodes (Gal *et al.*, 2005; Adhikari *et al.*, 2010; Thorne *et al.*, 2014). Two TPS genes were abundantly expressed in both cold and desiccation treated *D. destructor*. However, exposure to desiccation and cold did not lead to marked changes in the expression levels of aquaporin genes in *D. destructor*.

Some genes that did not show any relationship with nematode temperature or dessication tolerance in former reports were found significantly higher expressed in the cold or dessication treated *D. destructor* nematodes, such as genes encoding NLP-29 protein, mucins, UDP-glucuronosyltransferases (UGTs). The NLP-29 protein plays a role in response to physical injury and osmotic stress in *C. elegans* (Pujol *et al.*, 2008a,b). In *D. destructor*, NLP-29 was up-regulated in response to cold stress, suggesting that NLP-29 may be involved in the cold survival of the nematodes. Mucins have a direct role in combating pathogens and parasites in mice and are considered as important parts in the coordinated immune response (Hasnain *et al.*, 2013). Parsons *et al.* (2014) reported that mucin protects *C. elegans* against gram negative bacteria. Our study suggested that mucins may not only protect nematodes from biotic stressors, but may also provide protection against abiotic stressors, such as cold and desiccation. Certainly, further evidence is required to support the role for mucins in *D. destructor*.

The serine/threonine phosphatases proteins (STPs) have critical functions in the regulation of adaptive stress responses in plants, such as salt, drought, and cold stress (País *et al.*, 2009; Cui *et al.*, 2013). In animals, STPs play extremely diverse functions, such as sperm motility, cellular survival, and development and stress response (Lu *et al.*, 2007; Kolupaeva, 2019). In nematodes, such as *C. elegans*, *Trichostrongylus colubriformis* and *T. vitrinus*, STPs are involved in signal transduction or transcriptional activation and play key roles in development, reproductive, and survival (Barford *et al.*, 1998; Cantacessi *et al.*, 2010; Campbell *et al.*, 2011). This study showed that unigenes encoding STPs are up-regulated during cold stress of *D. destructor*. It is possible that STPs play a role in the cold survival of nematodes, as they are thought to do in plants.

UDP-glucose:glycoprotein glucosyltransferase (UGT) are enzymes of a detoxification system and are important for the maintenance of organism health (Laing *et al.*, 2010). The expression of UDP-glucose:glycoprotein glucosyltransferase was induced by temperature stress in *Schizosaccharomyces pombe* (Fernandez *et al.*, 1996). The induction of UGTs unigenes by cold and desiccation in *D. destructor* suggest that these unigenes may be stress related. Desiccation stress induction of ubiquitin genes occurs in Antarctic nematode (Adhikari *et al.*, 2009). The ubiquitin C-terminal hydrolase was reported to maintain osmotic balance and execute actin-dependent processes in the early *C. elegans* embryo (Kaitna *et al.*, 2002). Consistent with these observations, unigenes encoding ubiquitin-related proteins were up-regulated during desiccation stress of *D. destructor*. Cold and desiccation tolerance may elicit similar responses to injury during periods of extreme environmental conditions (Adhikari and Adams, 2011). In *D. destructor*, some genes were highly expressed in response to both of cold and desiccation stress, such as genes encoding proteins involved in mucin 5B, gut esterase, and protein DHS-7. By contrast, some genes were only activated during cold or desiccation in *D. destructor*. There was also a large number of unigenes that were down-regulated in the stress treated nematodes, such as genes encoding protein HSP-12.2, aldolase, nose resistant to fluoxetine protein 6 (NRF6). Expression levels of aldolase were reduced in response to heat stress in maize (Duke and Doehlert, 1996). The NRF protein is associated with drug and yolk transport across the basal intestinal membrane surface in *C. elegans* (Choy *et al.*, 2005). Our results show that *D. destructor* actively responds to stress via expression of a series of genes include the above genes. However, how these up/down-regulated genes regulate cold and desiccation tolerance in *D. destructor* requires further studies.

*Ditylenchus destructor* survives low temperature and desiccation for a long period without food (Lin *et al.*, 1997). Nematodes respond to stress in a complex fashion, with many transcriptional changes that include up- or down-regulated DEGs or pathways. Functional annotations of DEGs make it possible to know the molecular mechanisms underlying specific biological processes of different treated nematodes. However, some of the unigenes had no known homolog in any nematode or any other existing organisms. New genes or molecular markers related to different stages formation might be identified from this group in the future. We compared the cold-treated and desiccation treated groups to the untreated nematodes and found some similar responses. Further investigations, for example, correlation between desiccation and freeze tolerance, will be assessed in further studies.

Differentially expressed genes that include up-regulated and down-regulated genes were subjected to qRT-PCR analysis to validate the accuracy and reproducibility of the Illumina RNA-seq results. The selected genes encoding heat shock proteins, antifreeze proteins, and CRE-FAT-6 were reported as nematode stress-related genes. The expression results of RNA-Seq and qRT-PCR showed good consistency for all unigenes, which increases confidence in the RNA-seq. In each sample, billions of nematodes were pooled for RNA-seq. Different expressed genes were obtained by comparing of different groups. The results of the group change tendency are representative.

Knockdown of the unigenes 15628 and 15596 led to a decreased ability of the nematode’s cold tolerance. While, RNAi of the unigenes 15628 and 15596 in *D. destructor* produced no difference in nematode survival with respect to controls before exposure to cold conditions, mortality increased in the treated nematodes after exposure. The unigenes 15628 and 15596 were annotated as gut esterase 1*(ges-1)* and antifreezing protein, respectively.

The gut-specific esterase gene *(ges-1)* is normally expressed in the intestine of *C. elegans* (Kennedy *et al.*, 1993). *C. elegans ges-1* gene expression is dependent on WGATAR sites, and deletion of a tandem pair of WGATAR sites abolishes *ges-1* expression in the gut, but simultaneously activates *ges-1* expression in both the anterior and posterior pharynx, as well as in the rectum (Fukushige *et al.*, 2005). *ges-1* expression is responsible for approximately half of the total esterase activity in worms (McGhee *et al.*, 1990) and likely has important roles in the metabolism of exogenous molecules and nutrients (Egan *et al.*, 1995). Burns *et al.* (2017) reported that GES-1 hydrolyzes the nematicide wact-86, which is lethal to *C*. *elegans*. Marshall and McGhee (2001) suggest that pharyngeal expression of *ges-1* is advantageous only under certain developmental or environmental conditions in *C. briggsae*.

Antifreeze proteins assist nematode cold tolerance by freeze avoidance and cryoprotective dehydration (Wharton, 2011). Adhikari *et al.* (2010) reported that the *afp* gene was significantly up-regulated during exposure and recovery from freezing in *P. murrayi.* Our studies showed the important role of the unigenes 15628 and 15596 in response to cold conditions. However, there are open question. How is genes expression coordinated between the different components of the organ system? Is expression of these genes modulated by other transcription factors? What are the details for the physiological functions of the antifreeze proteins in *D. destructor*? Future investigation is required to unravel the functions of these genes.

This study provides the first insights into the cold and desiccation induced transcriptome of *D. destructor*. We did not use genomic data (Zheng *et al.*, 2016) because of insufficiency in assembly level of `Contig’ and annotation information. The *de novo* transcriptome assembly could provide a reliably results (Grabherr *et al.*, 2011; Haas *et al.*, 2013). We investigated the molecular characteristics of cold and desiccation treated nematodes and showed that the tolerance process involves complex traits. These findings provide a substantial contribution to existing sequence resources of *D. destructor*, and the annotated transcriptome sequences and gene expression profiles of *D. destructor* will contribute to explorations of the molecular mechanisms of environmental adaptation and the development of new effective control strategies.

## Supplementary material

The following online material is available for this article:

Figure S1 - Functional distribution of GO annotation and KEGG annotation.

Figure S2 - Volcano plots representing the differentially expressed genes (up and down-regulated) in different treated samples as compared to the control nematodes.

Figure S3 - *Ditylenchus destructor* in different conditions.

Table S1 - List of primers used in the study.

Table S2 - List and features of SSRs.

Table S3 - Statistic of SNPs that validated in all samples.

Table S4 - Genes highly expressed in both cold and desiccation treated nematodes.

Table S5 - Significantly enriched GO terms in the treated samples.

Table S6 - Significantly enriched KEGG pathways in the treated samples.
