# Supplementary material for: Transcriptome analyses of Ditylenchus destructor in responses to cold and desiccation stress
Source: Genet Mol Biol. 2020 Mar 23;43(1):e20180057. doi: 10.1590/1678-4685-GMB-2018-0057 (PMC7198036; doi:10.1590/1678-4685-GMB-2018-0057)
Supplement: Supplementary file 1 [file 1415-4757-GMB-43-1-e20180057-s4.pdf]

Supplementary Material to "Transcriptome analyses of *Ditylenchus destructor* in responses to cold and desiccation stress"

Table S1 - List of primers used in the study.

List of primers for qRT-PCR			
Unigenes	Annotation	Forward(5'-3')	Reverse(5'-3')
Actin		AGGCTGTGCTTTCGCTCTAC	GATTTCACGTTCTGCGGTGG
4783	small heat shock protein 12.6	CGCCGAATGAAATCGAGGTG	ACGACTCTTGCCGGTTTCTG
CL3560	calreticulin family protein	TCGGATCTCCAAGCAACGTC	TTCCCTCAAATCGGAAGGGC
CL11535	digestive system	GGCAAAGGAATCGAGAACGC	TCGGGATTCTGCACGCTTAG
CL2640	trichohyalin	CAAACTGAGCACCCGAGACA	ACTTCAGCCGTAACCGAGTC
Unigene20945	metabolism of cofactors and vitamins	TCATTTGTGTGAGGCGACGA	AAGGAGCGGCGATGATTAGG
CL6038	single-organism process	TCGAAAGGCCCAATAGAGCG	CAAAACTGCCTCCGAACGAC
Unigene15628	gut esterase 1	GCAATTCCTTGCTACCGTCG	CCATCCAAGCTCACGGAAGA
CL2302	Protein DHS-7	TCGGGAATCGGAGTGGAAAC	AGTGAATCCTTTGGCCTCGC
CL8617	Lipid metabolism	TTGTGGATTGGCACTTTGGC	TCCGGCAGCTTTTACGAAGA
Unigene15596	Antifreeze protein	CTGGTTTGACCGGATGGTGA	AAGGTTTTTGGAACCGCTGC
Unigene20352	UDP-glucuronosyl containing protein	GGTGTCGAAAATATGCCGCC	TATCGGTGGCCTTGGATTGG
CL11742	hypothetical protein SINV_11101	CGAGTTGGGGTAACTGCTGA	ATCCTCCATCGGCCACAATC
Unigene15619	ubiquitin protein 1	ACCGTGAACCAGCCTTCAAA	TGTAGTCCGAGAGAGTGCGA
CL4446	GNS1 SUR4 membrane protein domain	GCCAAAGTGAGGTTCATGGC	TTATGCACGGATGGGTGTCC
49558	heat shock protein 20	GGCACTTTTGTTTGGCACTG	AACTGTATTTTGGGCGACTGAC
44796	heat shock protein 40	CTCAATCAGGATCGCAACACTT	GGAAATCGGACATGGAACTCTAT
44608	heat shock protein 90	GCAGACCAAGACGACAAAACC	GTCAGCACTCGATGAAGCAACT
C40042	CRE-FAT-6 protein	ATCTTGGGGTGTTTCTTCGTT	GCTCATCGGCTATGGTCTCA
C50356	FMRFamide-like peptide 11	AATCATTGTAGCCAGAACCGAC	TTCCATCCAAATCAAGTCACAG

Primers for amplification of genes for dsRNA production			
Gene name	Primer name	Primer sequence (5' to 3')	Amplicon size (bp)
Unigene15596	T7GUT-F	TAATACGACTCACTATAGGTCCCAAGTTGAAACCTCCT	371
	T7GUT-R	TAATACGACTCACTATAGGCAGAGCAAACTGCGAATGA	
Unigene15628	T7U96-F	TAATACGACTCACTATAGGGTGCTCCGAGTATTGACAGGC	380
	T7U96-R	TAATACGACTCACTATAGGGGAAACCGTAGGATTCGTCAGT	
Gfp	Gfp-F	TAATACGACTCACTATAGGTCCCAAGTTGAAACCTCCT	356
	Gfp-R	TAATACGACTCACTATAGGGAAGTTCACCTTGATGCCGTT	
