# Supplementary material for: Transcriptome analyses of Ditylenchus destructor in responses to cold and desiccation stress
Source: Genet Mol Biol. 2020 Mar 23;43(1):e20180057. doi: 10.1590/1678-4685-GMB-2018-0057 (PMC7198036; doi:10.1590/1678-4685-GMB-2018-0057)
Supplement: Supplementary file 2 [file 1415-4757-GMB-43-1-e20180057-s1.pdf]

Supplementary Material to "Transcriptome analyses of *Ditylenchus destructor* in responses to cold and desiccation stress"

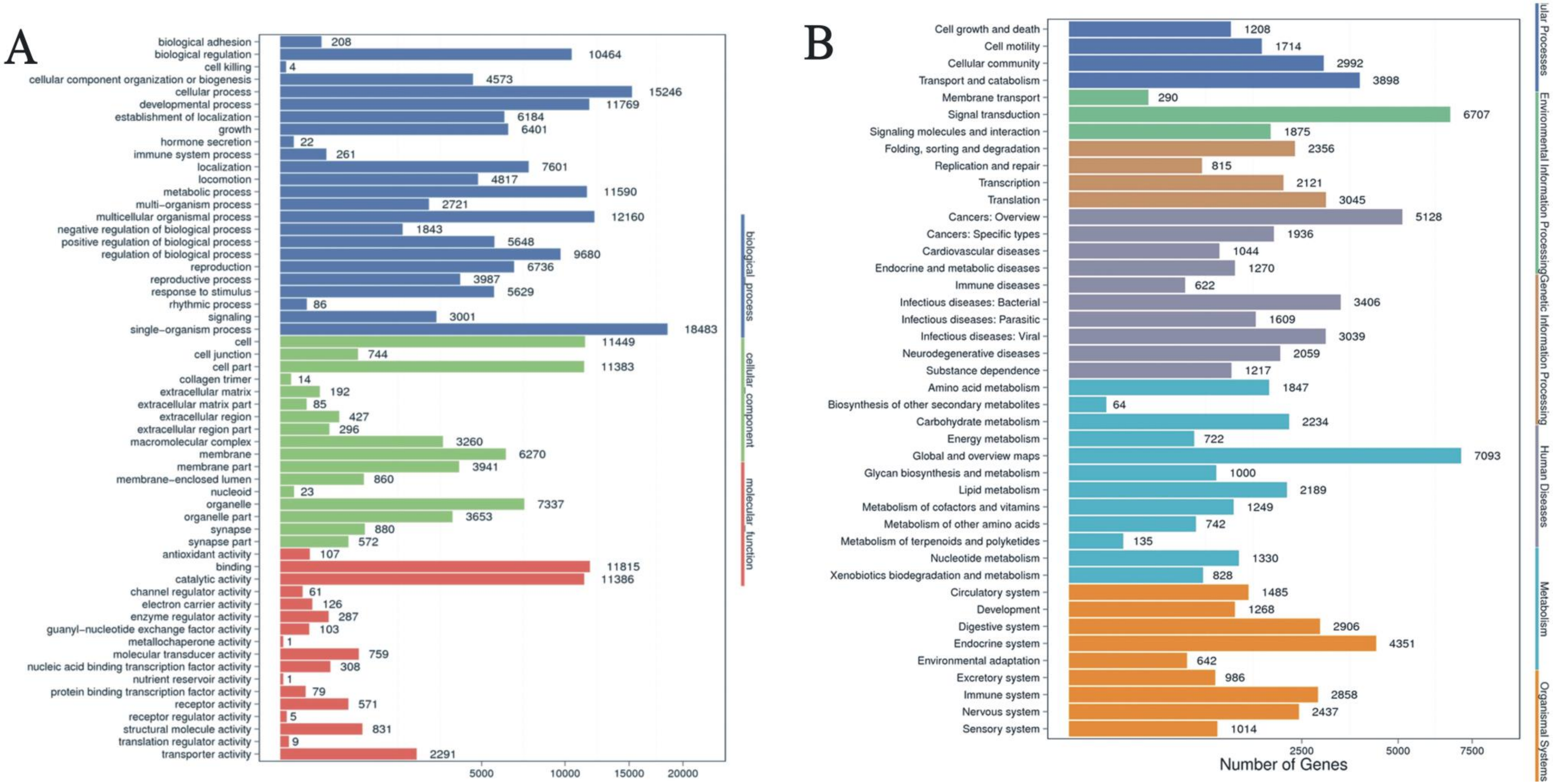

Figure S1 - Functional distribution of GO annotation (A) and KEGG annotation (B). X axis represents the number of Unigenes. Y axis represents the GO functional category/KEGG functional category.
