# Supplementary material for: Transcriptome analyses of Ditylenchus destructor in responses to cold and desiccation stress
Source: Genet Mol Biol. 2020 Mar 23;43(1):e20180057. doi: 10.1590/1678-4685-GMB-2018-0057 (PMC7198036; doi:10.1590/1678-4685-GMB-2018-0057)
Supplement: Supplementary file 5 [file 1415-4757-GMB-43-1-e20180057-s7.pdf]

Supplementary Material to "Transcriptome analyses of *Ditylenchus destructor* in responses to cold and desiccation stress"

Table S4 - Genes highly expressed in cold and desiccation treated nematodes. Unigenes tested by qPCR are shown in bold.

Gene ID	CK_6 (FPKM)	D_1G (FPKM)	D_2G (FPKM)	D_1W (FPKM)	D_2W (FPKM)	Nr	KEGG
**CL11535**	1.8	265.5	154.3	1112.4	789.9	gi|597891109/hypothetical protein Y032_0658g1253 [*Ancylostoma ceylanicum*]	bta:789503/3e-10/MUC5B, MUC5AC, mucin-5B; oligomeric mucus/gel-forming
Unigene15595	1.1	28.1	3.1	360.2	401.8	gi|557244171| /serine/threonine protein phosphatase, putative [*Eimeria necatrix*]	NA
Unigene15189	1.0	18.1	1.4	243.4	296.3	gi|557198975/hypothetical protein EMH_0013710 [*Eimeria mitis*]	NA
**Unigene15596**	5.5	100.3	4.8	1099.0	1219.3	gi|557234804/hypothetical protein EBH_0049890 [*Eimeria brunetti]*	NA
CL2646	1.1	1.0	5.1	147.7	159.6	gi|470260102/hypothetical protein DFA_04287 [*Dictyostelium fasciculatum]*	ame:724159/1e-08/GB10093; uncharacterized LOC724159; K11306 histone acetyltransferase MYST4
Unigene18439	2.1	8.9	23.9	158.9	126.3	NA	NA
Unigene15597	21.4	110.0	27.6	1328.5	1619.6	gi|557169829/myosin heavy chain, putative [*Eimeria praecox*]	cqu:CpipJ_CPIJ002507/3e-06/nuclear receptor co-repressor 1; K04650 nuclear receptor co-repressor 1
CL7558	1.8	4.2	1.8	111.6	74.6	NA	NA
CL6859	2.2	8.3	5.2	121.7	52.4	gi|341900824/hypothetical protein CAEBREN_18196 [*Caenorhabditis brenneri]*	fab:101814166/8e-08/HOXD3; homeobox D3
**Unigene20352**	2.5	6.6	9.2	132.9	102.8	gi|597892762/hypothetical protein Y032_0587g341 [*Ancylostoma ceylanicum*]	cbr:CBG09373/5e-82/Cbr-ugt-48 C. briggsae; K00699 glucuronosyltransferase

**Gene ID**	**CK_6 (FPKM)**	**D_1G (FPKM)**	**D_2G (FPKM)**	**D_1W (FPKM)**	**D_2W (FPKM)**	**Nr**	**KEGG**
CL5442	1.0	23.8	13.4	8.9	9.2	gi|541042271/udp-glucuronosyltransferase ugt-48 [*Ascaris suum*]	bmy:Bm1_13480/3e-53/UDP-glucoronosyl and UDP-glucosyl transferase family protein
CL3923	1.3	6.0	4.2	59.5	39.5	gi|25149822/Protein MIG-6, isoform c [*Caenorhabditis elegans*]	ocu:100344817/8e-93/ADAMTS18; K08632 a disintegrin and metalloproteinase with thrombospondin motifs 18
Unigene15564	1.9	217.9	5.3	85.5	47.7	gi|187027406/Protein CBG15126 [*Caenorhabditis briggsae*]	cfr:102511432/1e-13/FAM98B; family with sequence similarity 98, member B
Unigene9153	2.3	5.8	3.0	91.1	109.5	gi|55716698/hypothetical protein EMH_0033040 [*Eimeria mitis*]	mmu:218695/6e-09/Gm10044; K17299 POTE ankyrin domain family protein
Unigene3174	12.5	32.3	130.8	459.8	337.9	gi|557228633/hypothetical protein EPH_0028000 [*Eimeria praecox*]	dre:402861/7e-07/rab11fip1b, K12484 Rab11 family-interacting protein 1/2/5
CL2640.Ctig2	43.0	169.7	261.7	1541.3	1656.6	gi|123431955/trichohyalin [*Trichomonas vaginalis* G3]	dwi:Dwil_GK19899/1e-29/GK19899 gene product from transcript GK19899-RA; K18626 trichohyalin
**CL2640**	39.2	176.6	256.0	1261.6	1385.6	gi|449709444/trichohyalin, putative [*Entamoeba histolytica* KU27]	shr:100933589/1e-25/TCHH; trichohyalin
CL7250	19.1	70.0	43.6	613.9	446.3	gi|637345608/PREDICTED: glycine-rich cell wall structural protein [*Anolis carolinensis*]	cin:100182977/5e-20/heterogeneous nuclear ribonucleoprotein A3 homolog 2-like
CL757	1.5	230.8	14.6	2.8	8.0	NA	NA
**CL4446**	26.4	1185.3	120.6	30.1	25.0	gi|560135252/GNS1 SUR4 membrane protein domain containing protein [*Haemonchus contortus*]	xma:102231386/8e-41/elongation of very long chain fatty acids protein 6-like; K10203 [EC:2.3.1.199]
Unigene10683	1.0	40.3	4.6	8.6	1.8	gi|187027406/Protein CBG15126 [*Caenorhabditis briggsae]*	cin:100185025/7e-10/heterogeneous nuclear ribonucleoprotein D-like; K13044
CL566	1.0	10.7	25.5	0.0	0.0	gi|170591100/GMP synthase [*Brugia malayi*]	bmy:Bm1_44235/0.0/GMP synthase; K01951

Gene ID	CK_6 (FPKM)	D_1G (FPKM)	D_2G (FPKM)	D_1W (FPKM)	D_2W (FPKM)	Nr	KEGG
CL2521	2.3	103.7	53.6	12.8	11.4	gi|556759154/PREDICTED: uncharacterized protein YIR042C-like [*Pantholops hodgsonii*]	NA
CL1727	2.2	26.0	34.3	5.3	5.7	gi|597896822/hypothetical protein Y032_0446g1597 [*Ancylostoma ceylanicum*]	cel:CELE_T22H6.2/2e-99/alh-13; ALdehyde deHydrogenase; K12657 delta-1-pyrroline- 5-carboxylate synthetase [EC:2.7.2.11 1.2.1.41]
CL3801	1.3	18.5	20.0	0.0	0.0	gi|393907986/CBR-NURF-1 protein [*Loa loa*]	bmy:Bm1_11520/3e-46/PHD-finger family protein; K11728 nucleosome-remodeling factor subunit BPTF
**Unigene15619**	2.8	27.4	40.0	15.6	17.4	gi|341879718/hypothetical protein CAEBREN_32771 [*Caenorhabditis brenneri*]	loa:LOAG_06978/8e-29/ubiquitin C II; K08770 ubiquitin C
Unigene15620	1.78	15.72	16.88	1.68	3.71	gi|542249255|PREDICTED: polyubiquitin-C-like [*Oreochromis niloticus*]	fab:101807900/3e-16/UBC; ubiquitin C; K08770
CL5442	1.0	23.8	13.4	8.9	9.2	gi|541042271/udp-glucuronosyltransferase ugt-48 [*Ascaris suum*]	bmy:Bm1_13480/3e-53/UDP-glucoronosyl and UDP-glucosyl transferase family protein; K00699 glucuronosyltransferase [EC:2.4.1.17]
CL757	1.5	230.8	14.6	2.8	8.0	NA	NA
CL10164	2.0	43.1	17.1	13.9	3.7	gi|3085018790/CRE-GPDH-2 protein [*Caenorhabditis remanei*]	cel:CELE_K11H3.1/5e-149/gpdh-2; Glycerol-3-Phosphate DeHydrogenase; K00006 [EC:1.1.1.8]
CL583	16.9	381.5	142.2	10.3	10.2	gi|597833081/hypothetical protein Y032_0351g3245 [*Ancylostoma ceylanicum*]	cel:CELE_K09H11.7/2e-121/K09H11.7; K19269 phosphoglycolate phosphatase [EC:3.1.3.18 3.1.3.48]
CL6083	77.5	1750.9	571.6	35.6	58.4	gi|225712408/Hydroxyacyl-coenzyme A dehydrogenase, mitochondrial precursor [*Lepeophtheirus salmonis*]	aqu:100637276/2e-81/hydroxyacyl-coenzyme A dehydrogenase, mitochondrial-like; K00022 3-hydroxyacyl-CoA dehydrogenase [EC:1.1.1.35]
**CL2302**	3.5	45.0	25.5	30.7	33.4	gi|597836309/hypothetical protein Y032_0287g1432 [*Ancylostoma ceylanicum*]	crg:105332293/5e-43/WW domain-containing oxidoreductase-like

Gene ID	CK_6 (FPKM)	D_1G (FPKM)	D_2G (FPKM)	D_1W (FPKM)	D_2W (FPKM)	Nr	KEGG
CL11535	1.8	265.5	154.3	1112.4	789.9	gi|597891109/hypothetical protein Y032_0658g1253 [*Ancylostoma ceylanicum*]	bta:789503/3e-10/MUC5B, MUC5AC, mucin-5B; oligomeric mucus/gel-forming;
Unigene3174	12.5	32.3	130.8	459.8	337.9	gi|557228633/hypothetical protein EPH_0028000 [*Eimeria praecox*]	dre:402861/7e-07/rab11fip1b, si:ch211-187d7.3; RAB11 family interacting protein 1 (class I) b;
Unigene15189	1.0	18.1	1.4	243.4	296.3	gi|557198975/hypothetical protein EMH_0013710 [*Eimeria mitis*]	NA
CL2554	13.7	266.0	67.5	114.1	99.4	gi|560120930/Protein F55H12.4 [*Haemonchus contortus*]	NA
Unigene15564	1.9	217.9	5.3	85.5	47.7	gi|187027406/Protein CBG15126 [*Caenorhabditis briggsae*]	cfr:102511432/1e-13/FAM98B; family with sequence similarity 98, member B; K15434 protein FAM98B
**Unigene20945**	4.3	77.0	24.2	73.7	110.3	gi|170317959/PLP synthase [*Heterodera glycines*]	cin:100179166/6e-83/probable pyridoxine biosynthesis SNZERR; K06215 pyridoxal 5'-phosphate synthase pdxS subunit [EC:4.3.3.6]
CL2302	6.1	114.3	53.3	66.5	85.0	gi|17532791/Protein DHS-7 [*Caenorhabditis elegans*]	crg:105332293/7e-51/WW domain-containing oxidoreductase-like; K19329
CL5833	4.0	10.3	2.9	52.3	57.7	gi|541041775/putative oxidoreductase dhs-27 [*Ascaris suum*]	NA
**Unigene15628**	1.5	27.9	5.2	39.6	25.1	gi|541044223/gut esterase 1 [*Ascaris suum*]	cel:CELE_R12A1.4/9e-84/ges-1; Gut esterase 1; K01044 carboxylesterase 1 [EC:3.1.1.1]
Unigene6241	2.7	49.3	16.1	28.8	31.5	gi|341879600/hypothetical protein CAEBREN_00122 [*Caenorhabditis brenneri*]	cbr:CBG13096/7e-12/Hypothetical protein CBG13096; K19329 WW domain-containing oxidoreductase
CL4901	1.2	25.5	7.6	15.4	8.3	NA	NA
**CL11742**	1.1	18.7	5.7	13.0	12.7	gi|322789360/hypothetical protein SINV_11101 [*Solenopsis invicta*]	bmor:101737103/7e-29/L-xylulose reductase-like; K03331 L-xylulose reductase [EC:1.1.1.10]

Gene ID	CK_6 (FPKM)	D_1G (FPKM)	D_2G (FPKM)	D_1W (FPKM)	D_2W (FPKM)	Nr	KEGG
CL4572	1.3	28.9	23.1	12.5	6.3	gi|340375388/PREDICTED: trans-1,2-dihydrobenzene-1,2-diol dehydrogenase-like [*Amphimedon queenslandica*]	xtr:100145807/1e-28/dhdh;; K00078 dihydrodiol dehydrogenase / D-xylose 1-dehydrogenase (NADP) [EC:1.3.1.20 1.1.1.179]
Unigene10683	1.0	40.3	4.6	8.6	1.8	gi|187027406/Protein CBG15126 [*Caenorhabditis briggsae*]	cin:100185025/7e-10/heterogeneous nuclear ribonucleoprotein D-like
CL9198	33.5	64.6	113.6	57.1	90.4	gi|74776553/RecName: alpha-trehalose-phosphate synthase [UDP-forming] 2;	bmor:101741435/6e-43/alpha-trehalose-phosphate synthase [UDP-forming]
CL3586	445.2	262.3	1641.8	1425.6	1125.7	gi|392920154/Protein LEA-1, isoform a [*Caenorhabditis elegans*]	NA
CL10174	285.5	1501.3	1269.1	471.0	1052.8	gi|24418520/RecName: Full= Protein LEA-1	NA
**4783**	499.1	57.6	36.9	122.2	24.7	gi|541039287/heat shock protein [*Ascaris suum]*	dvi:Dvir_GJ21096/7e-16/GJ21096 gene product from transcript GJ21096-RA; K09542 crystallin, alpha B
**CL3560**	2	3.8	20.4	30.8	23.8	gi|312065685|calreticulin family protein [*Loa loa*]	loa:LOAG_00322/0.0/calreticulin family protein; K08054 calnexin
**CL8617**	2.2	14.4	18.3	21.8	23.9	gi|597870679| hypothetical protein Y032_0023g847 [*Ancylostoma ceylanicum*]	loa:LOAG_00382/1e-77/oxidoreductase; K00011 aldehyde reductase [EC:1.1.1.21]
**CL6038**	1.66	9.1	6.8	40.5	35.8	gi|541045808| neurocalcin delta [*Ascaris suum*]	NA
U5578	0.98	0	0	42.1	35.3	gi|17557246| Protein NLP-29 [*Caenorhabditis elegans*]	spu:594428/3e-11/peroxisomal membrane protein; K13344
CL10174	285.5	1501.3	471.0	1269.1	1052.8	gi|24418520| LEA1 protein [*Aphelenchus avenae*]	NA
CL8223	3.2	4.2	9.5	22.4	32.6	gi|24418520| LEA1 protein [*Aphelenchus avenae*]	ptg:102962717/7e-07/SRRM2; serine/arginine repetitive matrix 2; K13172
CL1306	459.0	496.4	1822.0	3630.2	2894.2	gi|392920154|Protein LEA-1, isoform a [*Caenorhabditis elegans*]	nvi:100122475/3e-19/microtubule-associated protein futsch-like; K10380

**Gene ID**	**CK_6 (FPKM)**	**D_1G (FPKM)**	**D_2G (FPKM)**	**D_1W (FPKM)**	**D_2W (FPKM)**	**Nr**	**KEGG**
CL2422	0.25	1.76	8.14	6.68	4.67	gi|90959527|PvLEA1 protein [*Polypedilum vanderplanki*]	sp|Q95V77|LEA1_APHAV
CL4616	3644.4	1731.6	15608.3	5381.9	12046.3	gi|146331045/LEA5 protein [*Steinernema carpocapsae]*	NA
**49558**	8.8	25.4	19.6	28	30.6	gu|130157.1|small HSP21-like protein [*Bursaphelenchus xylophilus*]	NA
CL653	724.5	1189.4	1033.9	297.7	389.6	gi|573006128|hsp 70A [*Ditylenchus destructor*]	bmy:Bm1_43675/0.0/heat shock 70 kDa protein
CL9628	1.9	4.1	4	4.9	4.0	gi|221706451|hsp 90 [*Steinernema feltiae*]	loa:LOAG_00073/8e-07/heat shock protein 90
U11847	1.7	2	1	46.7	49.9	gi|170591664|small heat shock protein 12.6, [*Brugia malayi*]	tgu:100230097/7e-13/CRYAB; crystallin, alpha B
CL9687	4.2	55.0	39.4	1.4	0.5	gi|328925282/heat shock protein 20 [*Bursaphelenchus doui*]	NA
CL9198	33.5	64.6	57.1	113.6	90.4	gi|74776553|Trehalose-6-phosphate synthase 2	bmor:101741435/6e-43/alpha,alpha-trehalose-phosphate synthase [UDP-forming];
Unigene13345	0.37	2.15	1.41	0.7	2.05	gi|74776556|Trehalose-6-phosphate synthase 1;	sp|Q5K2C4|TPS1_APHAV/0.0/Alpha,alpha-trehalose-phosphate synthase [UDP-forming] 1
CL1469	0	1.39	0	8.11	12.56	gi|541048301| serine threonine-protein phosphatase 5 [*Ascaris suum*]	bmy:Bm1_15955/0.0/serine/threonine protein phosphatase 5
CL1053	285	173.1	633.2	2256.3	1547.9	gi|514684442|response regulator receiver protein [Salpingoeca rosetta]	dre:402861/8e-23/ RAB11 family interacting protein 1 (class I) b
