# Supplementary material for: Transcriptome analyses of Ditylenchus destructor in responses to cold and desiccation stress
Source: Genet Mol Biol. 2020 Mar 23;43(1):e20180057. doi: 10.1590/1678-4685-GMB-2018-0057 (PMC7198036; doi:10.1590/1678-4685-GMB-2018-0057)
Supplement: Supplementary file 6 [file 1415-4757-GMB-43-1-e20180057-s2.pdf]

Supplementary Material to "Transcriptome analyses of *Ditylenchus destructor* in responses to cold and desiccation stress"

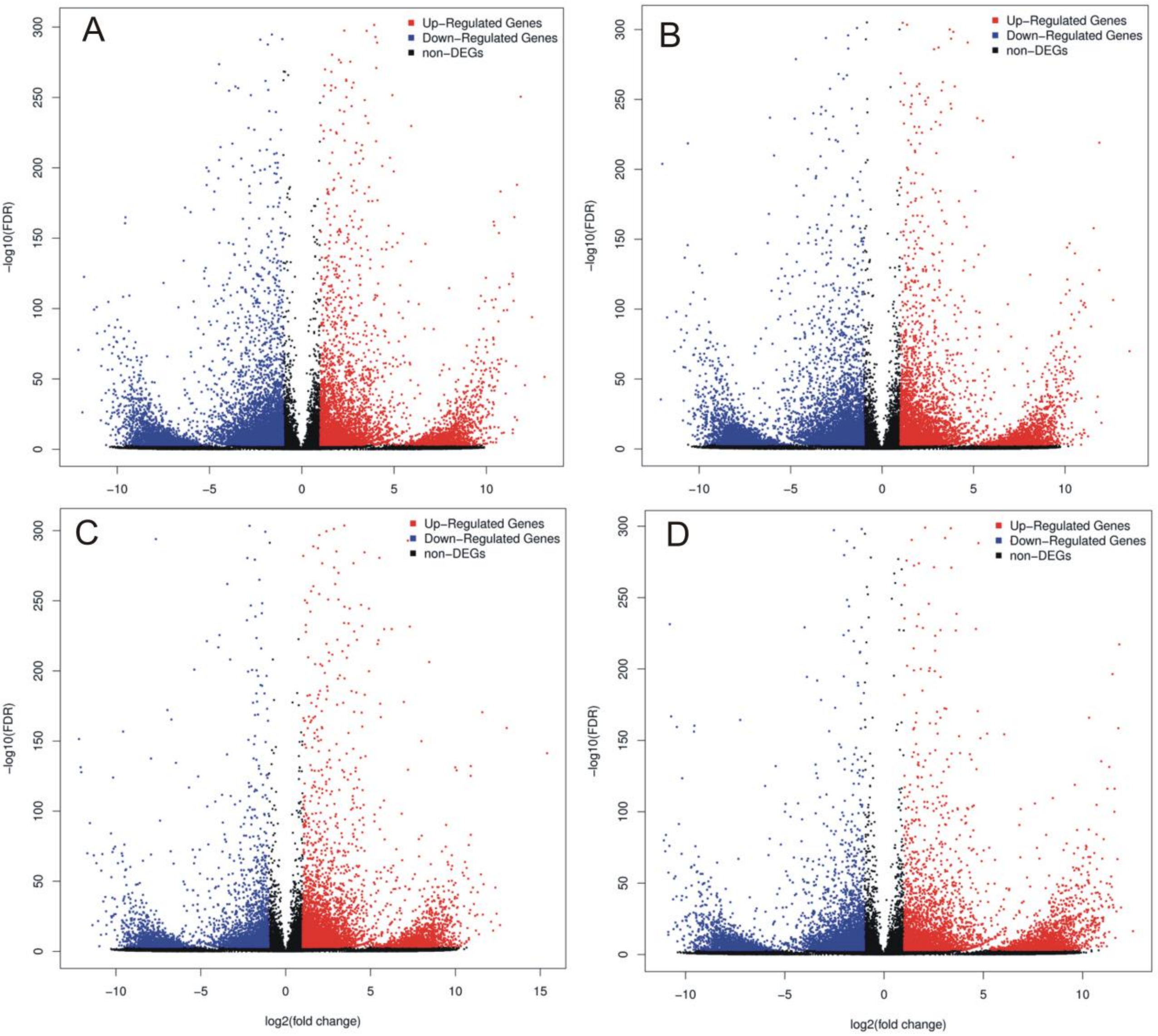

Figure S2 - Volcano plots representing the differentially expressed genes (up and down-regulated) in different treated samples as compared to the control nematodes. (A) CK *vs.* D_1W. (B) CK *vs.* D_2W. (C) CK *vs.* D_1G. (D) CK *vs.* D_2G. The X-axis means fold change of the gene between control and treated group. Y-axis represents Log10 transformed fold change. The significant DEGs were considered as FDR ≤0.01 and fold change ≥2.
