# Supplementary material for: Transcriptome analyses of Ditylenchus destructor in responses to cold and desiccation stress
Source: Genet Mol Biol. 2020 Mar 23;43(1):e20180057. doi: 10.1590/1678-4685-GMB-2018-0057 (PMC7198036; doi:10.1590/1678-4685-GMB-2018-0057)
Supplement: Supplementary file 7 [file 1415-4757-GMB-43-1-e20180057-s3.pdf]

Supplementary Material to "Transcriptome analyses of *Ditylenchus destructor* in responses to cold and desiccation stress"

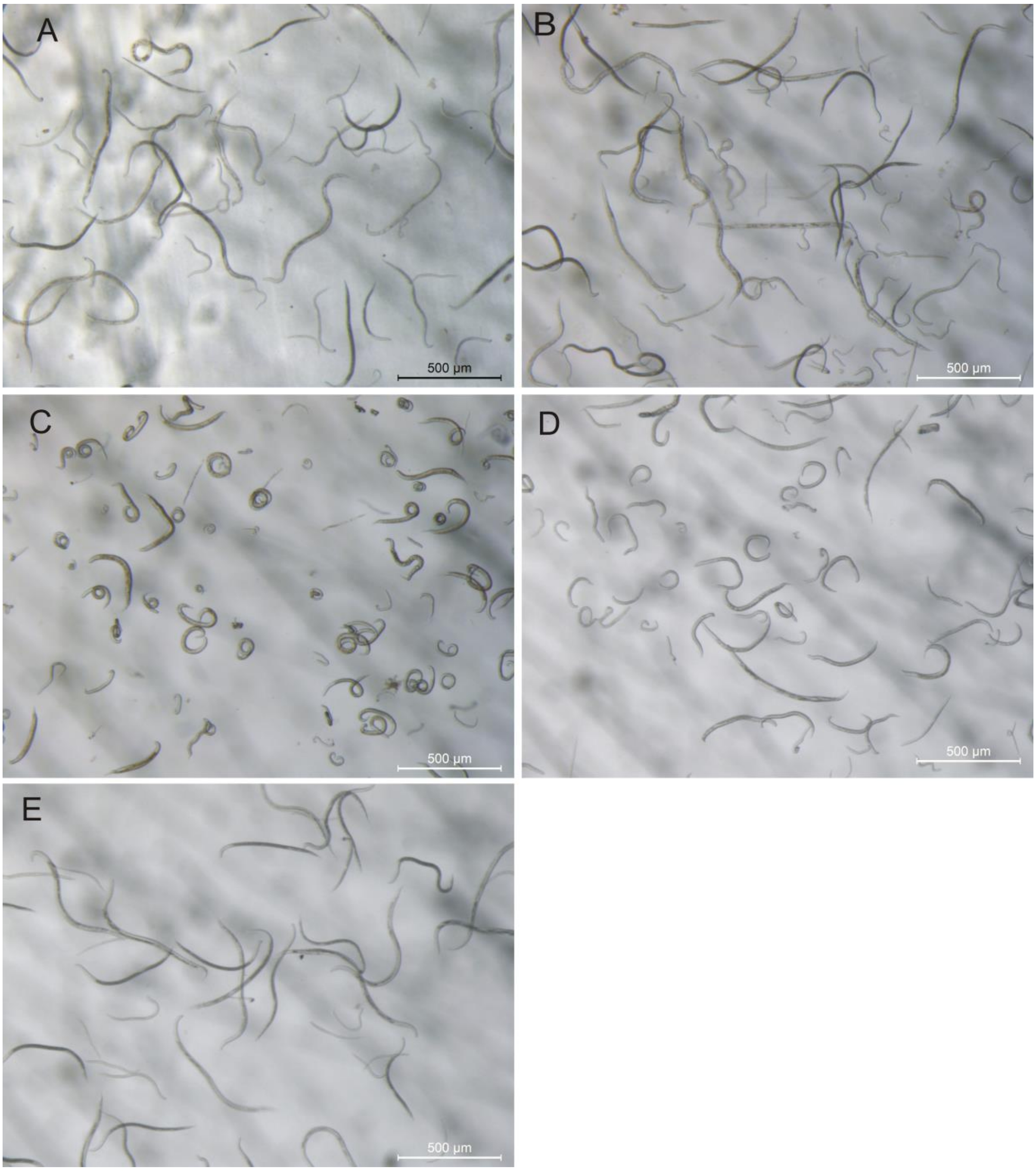

Figure S3 - *Ditylenchus destructor* in different conditions. (A) Nematodes exposed to water at -1°C for 35 days. (B) Nematodes exposed to water at -3°C for 35 days. (C) Nematodes exposed to 10% glycerol for 35 days. (D) Nematodes exposed to 20% glycerol for 5 days. (E) Non-treated nematodes in distilled water.
