# Supplementary material for: Transcriptome analyses of Ditylenchus destructor in responses to cold and desiccation stress
Source: Genet Mol Biol. 2020 Mar 23;43(1):e20180057. doi: 10.1590/1678-4685-GMB-2018-0057 (PMC7198036; doi:10.1590/1678-4685-GMB-2018-0057)
Supplement: Supplementary file 8 [file 1415-4757-GMB-43-1-e20180057-s8.pdf]

Supplementary Material to “Transcriptome analyses of *Ditylenchus destructor* in responses to cold and desiccation stress”

Table S5 – Significantly enriched GO terms in the treated samples.

	Gene Ontology term	Cluster frequency	Corrected P-value
**CK_6 *vs*. D_1W up-regulated**			
molecular_function	catalytic activity	1050 out of 1650, 63%	0.000
	alpha-1,3-mannosylglycoprotein 2-beta-N-acetylglucosaminyltransferase activity	9 out of 1650, 0.5%	0.000
	serine-type carboxypeptidase activity	8 out of 1650, 0.5%	0.003
	serine-type exopeptidase activity	9 out of 1650, 0.5%	0.005
cellular_component	peroxisome	24 out of 1117, 2.1%	0.001
	microbody	24 out of 1117, 2.1%	0.001
	MLL1/2 complex	7 out of 1117, 0.6%	0.012
	MLL1 complex	7 out of 1117, 0.6%	0.012
**CK_6 vs. D_1W down-regulated**			
molecular_function	ated channel activity	139 out of 2604, 5.3%	0.000
	ion channel activity	169 out of 2604, 6.5%	0.000
	substrate-specific channel activity	173 out of 2604, 6.6%	0.000
	channel activity	186 out of 2604, 7.1%	0.000
	passive transmembrane transporter activity	186 out of 2604, 7.1%	0.000
	transmembrane signaling receptor activity	97 out of 2604, 3.7%	0.003
	cation channel activity	96 out of 2604, 3.7%	0.009
	signal transducer activity	140 out of 2604, 5.4%	0.013
	molecular transducer activity	140 out of 2604, 5.4%	0.013
	extracellular ligand-gated ion channel activity	76 out of 2604, 2.9%	0.025
	receptor activity	109 out of 2604, 4.2%	0.025
	signaling receptor activity	102 out of 2604, 3.9%	0.038
	G-protein coupled receptor activity	53 out of 2604, 2.0%	0.043
	sugar-phosphatase activity	14 out of 2604, 0.5%	0.053
biological_process	carbohydrate catabolic process	46 out of 3152, 1.5%	0.026
	single-organism carbohydrate catabolic process	44 out of 3152, 1.4%	0.029
cellular_component	plasma membrane part	239 out of 1808, 13.2%	0.000
	synaptic membrane	80 out of 1808, 4.4%	0.001
	synapse	157 out of 1808, 8.7%	0.001
	plasma membrane	339 out of 1808, 18.8%	0.002
	cell periphery	362 out of 1808, 20.0%	0.005
	synapse part	106 out of 1808, 5.9%	0.010
	postsynaptic membrane	70 out of 1808, 3.9%	0.012

	Gene Ontology term	Cluster frequency	Corrected P-value
**CK_6 *vs.*D_2W up-regulated**			
molecular_function	serine-type carboxypeptidase activity	8 out of 1804, 0.4%	0.005
	alpha-1,3-mannosylglycoprotein 2-beta-N-acetylglucosaminyltransferase activity	8 out of 1804, 0.4%	0.011
	serine-type exopeptidase activity	9 out of 1804, 0.5%	0.011
biological_process	positive regulation of vacuole organization	5 out of 2178, 0.2%	0.014
	positive regulation of autophagic vacuole assembly	5 out of 2178, 0.2%	0.014
	developmental process	1166 out of 2178, 53.5%	0.035
	multicellular organismal development	1091 out of 2178, 50.1%	0.055
cellular_component	Cdc48p-Npl4p-Ufd1p AAA ATPase complex	6 out of 1245, 0.5%	0.049
**CK_6 *vs.*D_2W down-regulated**			
molecular_function	gated channel activity	141 out of 2445, 5.8%	0.000
	ion channel activity	170 out of 2445, 7.0%	0.000
	substrate-specific channel activity	174 out of 2445, 7.1%	0.000
	channel activity	185 out of 2445, 7.6%	0.000
	passive transmembrane transporter activity	185 out of 2445, 7.6%	0.000
	signal transducer activity	151 out of 2445, 6.2%	0.000
	molecular transducer activity	151 out of 2445, 6.2%	0.000
	cation channel activity	98 out of 2445, 4.0%	0.000
	transmembrane signaling receptor activity	97 out of 2445, 4.0%	0.000
	extracellular ligand-gated ion channel activity	78 out of 2445, 3.2%	0.001
	voltage-gated cation channel activity	46 out of 2445, 1.9%	0.001
	ligand-gated ion channel activity	89 out of 2445, 3.6%	0.001
	ligand-gated channel activity	89 out of 2445, 3.6%	0.001
	voltage-gated ion channel activity	48 out of 2445, 2.0%	0.002
	voltage-gated channel activity	48 out of 2445, 2.0%	0.002
	receptor activity	108 out of 2445, 4.4%	0.003
	signaling receptor activity	102 out of 2445, 4.2%	0.003
	G-protein coupled receptor activity	54 out of 2445, 2.2%	0.003
	ion transmembrane transporter activity	245 out of 2445, 10.0%	0.017
	neurotransmitter receptor activity	31 out of 2445, 1.3%	0.018
	acetylcholine receptor activity	23 out of 2445, 0.9%	0.026
	peptidyl-lysine 5-dioxygenase activity	7 out of 2445, 0.3%	0.027
biological_process	cell communication	408 out of 2935, 13.9%	0.000
	signaling	397 out of 2935, 13.5%	0.000
	single organism signaling	397 out of 2935, 13.5%	0.000
	synaptic transmission	122 out of 2935, 4.2%	0.000
	cell-cell signaling	129 out of 2935, 4.4%	0.000
	regulation of behavior	104 out of 2935, 3.5%	0.000
	regulation of locomotion	170 out of 2935, 5.8%	0.000
	regulation of response to stimulus	202 out of 2935, 6.9%	0.010
	single-organism carbohydrate catabolic process	43 out of 2935, 1.5%	0.011

	Gene Ontology term	Cluster frequency	Corrected P-value
	G-protein coupled receptor signaling pathway	85 out of 2935, 2.9%	0.016
	regulation of pharyngeal pumping	63 out of 2935, 2.1%	0.023
	regulation of feeding behavior	63 out of 2935, 2.1%	0.029
	peptidyl-lysine hydroxylation	7 out of 2935, 0.2%	0.031
	peptidyl-lysine hydroxylation to 5-hydroxy-L-lysine	7 out of 2935, 0.2%	0.031
	carbohydrate catabolic process	43 out of 2935, 1.5%	0.051
cellular_component	plasma membrane part	251 out of 1733, 14.5%	0.000
	synapse	171 out of 1733, 9.9%	0.000
	synapse part	119 out of 1733, 6.9%	0.000
	synaptic membrane	87 out of 1733, 5.0%	0.000
	plasma membrane	348 out of 1733, 20.1%	0.000
	cell periphery	373 out of 1733, 21.5%	0.000
	postsynaptic membrane	77 out of 1733, 4.4%	0.000
	neuron part	172 out of 1733, 9.9%	0.001
	neuron projection	147 out of 1733, 8.5%	0.002
	cell projection	182 out of 1733, 10.5%	0.003
	cation channel complex	35 out of 1733, 2.0%	0.004
	ion channel complex	43 out of 1733, 2.5%	0.018
	transmembrane transporter complex	43 out of 1733, 2.5%	0.039
	membrane	832 out of 1733, 48.0%	0.039
	striated muscle dense body	64 out of 1733, 3.7%	0.041
**CK_6 *vs.*D_1G up-regulated**			
molecular_function	stearoyl-CoA 9-desaturase activity	10 out of 1835, 0.5%	0.004
	acyl-CoA desaturase activity	10 out of 1835, 0.5%	0.004
	6-phosphofructo-2-kinase activity	12 out of 1835, 0.7%	0.007
	fructose-2,6-bisphosphate 2-phosphatase activity	12 out of 1835, 0.7%	0.007
	lyase activity	71 out of 1835, 3.9%	0.009
	phosphoglycerate dehydrogenase activity	6 out of 1835, 0.3%	0.010
	oxidoreductase activity, acting on paired donors, with oxidation of a pair of donors resulting in the reduction of molecular oxygen to two molecules of water	10 out of 1835, 0.5%	0.011
	catalytic activity	1138 out of 1835, 62.0%	0.016
	phosphofructokinase activity	14 out of 1835, 0.8%	0.017
	pyruvate kinase activity	6 out of 1835, 0.3%	0.027
	potassium ion binding	6 out of 1835, 0.3%	0.027
	alkali metal ion binding	6 out of 1835, 0.3%	0.027
	oxidoreductase activity, acting on CH-OH group of donors	55 out of 1835, 3.0%	0.027
	sugar-phosphatase activity	12 out of 1835, 0.7%	0.028
	MAP-kinase scaffold activity	8 out of 1835, 0.4%	0.040

	Gene Ontology term	Cluster frequency	Corrected P-value
	substrate-specific transmembrane transporter activity	199 out of 1835, 10.8%	0.049
	substrate-specific transporter activity	210 out of 1835, 11.4%	0.051
biological_process	organic acid biosynthetic process	68 out of 2315, 2.9%	0.000
	carboxylic acid biosynthetic process	68 out of 2315, 2.9%	0.000
	alpha-amino acid biosynthetic process	41 out of 2315, 1.8%	0.000
	small molecule biosynthetic process	76 out of 2315, 3.3%	0.000
	L-serine metabolic process	19 out of 2315, 0.8%	0.000
	cellular amino acid biosynthetic process	51 out of 2315, 2.2%	0.001
	unsaturated fatty acid biosynthetic process	11 out of 2315, 0.5%	0.001
	serine family amino acid biosynthetic process	21 out of 2315, 0.9%	0.002
	unsaturated fatty acid metabolic process	11 out of 2315, 0.5%	0.002
	serine family amino acid metabolic process	27 out of 2315, 1.2%	0.002
	monocarboxylic acid biosynthetic process	20 out of 2315, 0.9%	0.006
	single-organism biosynthetic process	179 out of 2315, 7.7%	0.010
	carboxylic acid metabolic process	132 out of 2315, 5.7%	0.011
	oxoacid metabolic process	137 out of 2315, 5.9%	0.022
	alpha-amino acid metabolic process	56 out of 2315, 2.4%	0.025
	organic acid metabolic process	137 out of 2315, 5.9%	0.026
	fatty acid biosynthetic process	16 out of 2315, 0.7%	0.027
	fructose metabolic process	12 out of 2315, 0.5%	0.036
	fructose 2,6-bisphosphate metabolic process	12 out of 2315, 0.5%	0.036
	L-serine biosynthetic process	6 out of 2315, 0.3%	0.041
	monocarboxylic acid metabolic process	48 out of 2315, 2.1%	0.049
	lipid metabolic process	106 out of 2315, 4.6%	0.051
	response to inorganic substance	38 out of 2315, 1.6%	
cellular_component	membrane	651 out of 1278, 50.9%	0.000
	membrane part	415 out of 1278, 32.5%	0.008
	integral component of membrane	312 out of 1278, 24.4%	0.023
	plasma membrane region	52 out of 1278, 4.1%	0.038
	intrinsic component of membrane	312 out of 1278, 24.4%	0.043
**CK_6 *vs.*D_1G down-regulated**			
molecular_function	monooxygenase activity	25 out of 1142, 2.2%	0.001
	extracellular-glutamate-gated ion channel activity	10 out of 1142, 0.9%	0.065
biological_process	L-phenylalanine metabolic process	9 out of 1373, 0.7%	0.005
	L-phenylalanine catabolic process	9 out of 1373, 0.7%	0.005
	erythrose 4-phosphate/phosphoenolpyruvate family amino acid metabolic process	9 out of 1373, 0.7%	0.005
	erythrose 4-phosphate/phosphoenolpyruvate family amino acid catabolic process	9 out of 1373, 0.7%	0.005
	long-chain fatty acid metabolic process	16 out of 1373, 1.2%	0.005
	tyrosine metabolic process	9 out of 1373, 0.7%	0.007

	Gene Ontology term	Cluster frequency	Corrected P-value
	alpha-amino acid catabolic process	20 out of 1373, 1.5%	0.033
	aromatic amino acid family catabolic process	11 out of 1373, 0.8%	0.043
cellular_component	host	3 out of 747, 0.4%	0.039
	host cell part	3 out of 747, 0.4%	0.039
	extraorganismal space	3 out of 747, 0.4%	0.039
	host cell	3 out of 747, 0.4%	0.039
	other organism	3 out of 747, 0.4%	0.039
	other organism cell	3 out of 747, 0.4%	0.039
	other organism part	3 out of 747, 0.4%	0.039
CK_6 ***vs.* D_2G** **up-regulated**			
molecular_function	structural constituent of ribosome	83 out of 1579, 5.3%	0.000
	RNA binding	96 out of 1579, 6.1%	0.000
	aryl sulfotransferase activity	7 out of 1579, 0.4%	0.001
	1-hydroxypyrene sulfotransferase activity	7 out of 1579, 0.4%	0.001
	translation initiation factor binding	5 out of 1579, 0.3%	0.010
	structural molecule activity	99 out of 1579, 6.3%	0.020
	Notch binding	9 out of 1579, 0.6%	0.032
	ribosomal large subunit binding	5 out of 1579, 0.3%	0.034
	transaminase activity	17 out of 1579, 1.1%	0.040
	transferase activity, transferring nitrogenous groups	17 out of 1579, 1.1%	0.040
biological_process	gene expression	228 out of 1947, 11.7%	0.000
	translation	123 out of 1947, 6.3%	0.000
	cellular macromolecule biosynthetic process	197 out of 1947, 10.1%	0.000
	macromolecule biosynthetic process	197 out of 1947, 10.1%	0.001
	translational initiation	28 out of 1947, 1.4%	0.005
	phagosome maturation involved in apoptotic cell clearance	18 out of 1947, 0.9%	0.008
	organic substance biosynthetic process	301 out of 1947, 15.5%	0.010
	viral gene expression	15 out of 1947, 0.8%	0.011
	viral transcription	15 out of 1947, 0.8%	0.011
	multi-organism metabolic process	15 out of 1947, 0.8%	0.011
	ribosome assembly	10 out of 1947, 0.5%	0.012
	viral process	19 out of 1947, 1.0%	0.015
	multi-organism cellular process	19 out of 1947, 1.0%	0.015
	viral life cycle	16 out of 1947, 0.8%	0.024
	translational termination	15 out of 1947, 0.8%	0.025
	erythrocyte homeostasis	8 out of 1947, 0.4%	0.029
	phagosome maturation	18 out of 1947, 0.9%	0.031
	positive regulation of organelle assembly	5 out of 1947, 0.3%	0.042
	cellular biosynthetic process	288 out of 1947, 14.8%	0.044

	**Gene Ontology term**	**Cluster frequency**	**Corrected P-value**
cellular_component	ribosome	90 out of 1111, 8.1%	0.000
	ribosomal subunit	53 out of 1111, 4.8%	0.000
	cytosolic ribosome	36 out of 1111, 3.2%	0.000
	ribonucleoprotein complex	130 out of 1111, 11.7%	0.000
	cytosolic part	40 out of 1111, 3.6%	0.000
	small ribosomal subunit	36 out of 1111, 3.2%	0.000
cellular_component	organelle	665 out of 1111, 59.9%	0.000
	intracellular organelle	651 out of 1111, 58.6%	0.000
	cytosolic small ribosomal subunit	24 out of 1111, 2.2%	0.000
	cytoplasmic part	454 out of 1111, 40.9%	0.000
	intracellular part	777 out of 1111, 69.9%	0.000
	intracellular	805 out of 1111, 72.5%	0.000
	intracellular membrane-bounded organelle	480 out of 1111, 43.2%	0.001
	membrane-bounded organelle	496 out of 1111, 44.6%	0.001
	phagocytic vesicle	16 out of 1111, 1.4%	0.011
	nucleolus	38 out of 1111, 3.4%	0.013
	cytoplasm	542 out of 1111, 48.8%	0.033
	mitochondrion	98 out of 1111, 8.8%	0.037
**CK_6 *vs*. D_2G down-regulated**			
molecular_function	transporter activity	272 out of 1690, 16.1%	0.000
	channel activity	136 out of 1690, 8.0%	0.000
	passive transmembrane transporter activity	136 out of 1690, 8.0%	0.000
	substrate-specific channel activity	124 out of 1690, 7.3%	0.000
	gated channel activity	99 out of 1690, 5.9%	0.000
	substrate-specific transporter activity	211 out of 1690, 12.5%	0.000
	ion channel activity	116 out of 1690, 6.9%	0.000
	substrate-specific transmembrane transporter activity	199 out of 1690, 11.8%	0.000
	ion transmembrane transporter activity	188 out of 1690, 11.1%	0.000
	transmembrane transporter activity	224 out of 1690, 13.3%	0.001
	transmembrane signaling receptor activity	71 out of 1690, 4.2%	0.001
	signal transducer activity	101 out of 1690, 6.0%	0.003
	molecular transducer activity	101 out of 1690, 6.0%	0.003
	voltage-gated cation channel activity	35 out of 1690, 2.1%	0.003
	potassium channel activity	40 out of 1690, 2.4%	0.004
	potassium ion transmembrane transporter activity	46 out of 1690, 2.7%	0.006
	cation channel activity	69 out of 1690, 4.1%	0.006
	signaling receptor activity	74 out of 1690, 4.4%	0.012
	receptor activity	78 out of 1690, 4.6%	0.014
	voltage-gated ion channel activity	35 out of 1690, 2.1%	0.017
	voltage-gated channel activity	35 out of 1690, 2.1%	0.017
	lactate dehydrogenase activity	6 out of 1690, 0.4%	0.036
	L-lactate dehydrogenase activity	6 out of 1690, 0.4%	0.036

	**Gene Ontology term**	**Cluster frequency**	**Corrected P-value**
	metal ion transmembrane transporter activity	84 out of 1690, 5.0%	0.051
biological_process	regulation of behavior	84 out of 2041, 4.1%	0.000
	regulation of pharyngeal pumping	54 out of 2041, 2.6%	0.000
	regulation of feeding behavior	54 out of 2041, 2.6%	0.000
	cell junction assembly	33 out of 2041, 1.6%	0.000
	cell junction organization	37 out of 2041, 1.8%	0.002
	ion transport	192 out of 2041, 9.4%	0.003
	larval somatic muscle development	6 out of 2041, 0.3%	0.005
	behavior	202 out of 2041, 9.9%	0.007
	gas transport	6 out of 2041, 0.3%	0.020
	oxygen transport	6 out of 2041, 0.3%	0.020
	G-protein coupled receptor signaling pathway	64 out of 2041, 3.1%	0.021
	regulation of response to stimulus	147 out of 2041, 7.2%	0.022
	somatic muscle development	6 out of 2041, 0.3%	0.055
cellular_component	synapse	119 out of 1232, 9.7%	0.000
	synapse part	85 out of 1232, 6.9%	0.000
	plasma membrane part	174 out of 1232, 14.1%	0.000
	plasma membrane	247 out of 1232, 20.0%	0.000
	cell periphery	265 out of 1232, 21.5%	0.000
	synaptic membrane	58 out of 1232, 4.7%	0.003
	postsynaptic membrane	53 out of 1232, 4.3%	0.007
	membrane	607 out of 1232, 49.3%	0.011
	cell junction	95 out of 1232, 7.7%	0.018
	neuron part	122 out of 1232, 9.9%	0.023
	axon part	15 out of 1232, 1.2%	0.028
	neuron projection	105 out of 1232, 8.5%	0.036
	cell-substrate junction	17 out of 1232, 1.4%	0.040
