# Supplementary material for: Transcriptome analyses of Ditylenchus destructor in responses to cold and desiccation stress
Source: Genet Mol Biol. 2020 Mar 23;43(1):e20180057. doi: 10.1590/1678-4685-GMB-2018-0057 (PMC7198036; doi:10.1590/1678-4685-GMB-2018-0057)
Supplement: Supplementary file 9 [file 1415-4757-GMB-43-1-e20180057-s9.pdf]

Supplementary Material to "Transcriptome analyses of *Ditylenchus destructor* in responses to cold and desiccation stress"

Table S6 – Significantly enriched KEGG pathways in the treated samples.

A: Up-regulated DEGs between CK_6 and D_1W

KEGGpathway	DEGs genes with pathway annotation (3747)	*P* value	Q value	KO ID
Peroxisome	87 (2.32%)	0.0000	0.0013	ko04146
Retinol metabolism	74 (1.97%)	0.0001	0.0173	ko00830
Thyroid hormone signaling pathway	142 (3.79%)	0.0002	0.0173	ko04919
PPAR signaling pathway	53 (1.41%)	0.0002	0.0173	ko03320
Glycerolipid metabolism	54 (1.44%)	0.0003	0.0173	ko00561
Spliceosome	154 (4.11%)	0.0003	0.0173	ko03040
Metabolic pathways	674 (17.99%)	0.0004	0.0173	ko01100
Shigellosis	108 (2.88%)	0.0006	0.0237	ko05131
Pentose and glucuronate interconversions	57 (1.52%)	0.0007	0.0251	ko00040
Bile secretion	76 (2.03%)	0.0009	0.0291	ko04976
Hematopoietic cell lineage	56 (1.49%)	0.0015	0.0449	ko04640
Drug metabolism - other enzymes	64 (1.71%)	0.0017	0.0449	ko00983
Fatty acid biosynthesis	13 (0.35%)	0.0018	0.0449	ko00061
Glycerophospholipid metabolism	66 (1.76%)	0.0019	0.0449	ko00564
Pathogenic Escherichia coli infection	98 (2.62%)	0.0025	0.0545	ko05130

B: Down-regulated DEGs between CK_6 and D_1W

KEGGpathway	DEGs genes with pathway annotation (5909)	*P* value	Q value	KO ID
Neuroactive ligand-receptor interaction	173 (2.93%)	0.0000	0.0009	ko04080
Calcium signaling pathway	142 (2.4%)	0.0001	0.0213	ko04020
Aldosterone synthesis and secretion	102 (1.73%)	0.0002	0.0213	ko04925
Nitrogen metabolism	25 (0.42%)	0.0004	0.0282	ko00910
Long-term potentiation	84 (1.42%)	0.0006	0.0282	ko04720
Fructose and mannose metabolism	53 (0.9%)	0.0006	0.0282	ko00051
D-Glutamine and D-glutamate metabolism	10 (0.17%)	0.0006	0.0282	ko00471
cGMP-PKG signaling pathway	167 (2.83%)	0.0010	0.0400	ko04022
Lipoic acid metabolism	9 (0.15%)	0.0013	0.0483	ko00785

C: Up-regulated DEGs between CK_6 and D_2W

KEGG pathway	DEGs genes with pathway annotation (3906)	*P* value	Q value	KO ID
Retinol metabolism	84 (2.15%)	0.0000	0.0007	ko00830
Pathogenic Escherichia coli infection	111 (2.84%)	0.0001	0.0099	ko05130
Pentose and glucuronate interconversions	62 (1.59%)	0.0001	0.0109	ko00040

Amoebiasis	120 (3.07%)	0.0001	0.0109	ko05146
Hypertrophic cardiomyopathy (HCM)	92 (2.36%)	0.0005	0.0287	ko05410
Tight junction	164 (4.2%)	0.0005	0.0287	ko04530
Dilated cardiomyopathy (DCM)	93 (2.38%)	0.0009	0.0401	ko05414
Spliceosome	156 (3.99%)	0.0010	0.0401	ko03040
Metabolism of xenobiotics by cytochrome P450	63 (1.61%)	0.0011	0.0401	ko00980
Shigellosis	109 (2.79%)	0.0016	0.0535	ko05131

D: Down-regulated DEGs between CK_6 and D_2W

KEGG pathway	DEGs genes with pathway annotation (5511)	*P* value	Q value	KO ID
Neuroactive ligand-receptor interaction	185 (3.36%)	0.0000	0.0000	ko04080
Fructose and mannose metabolism	59 (1.07%)	0.0000	0.0002	ko00051
Hippo signaling pathway - fly	85 (1.54%)	0.0000	0.0054	ko04391
Calcium signaling pathway	135 (2.45%)	0.0001	0.0069	ko04020
Phenylalanine, tyrosine and tryptophan biosynthesis	13 (0.24%)	0.0001	0.0078	ko00400
Lipoic acid metabolism	10 (0.18%)	0.0001	0.0078	ko00785
African trypanosomiasis	27 (0.49%)	0.0003	0.0156	ko05143
Long-term depression	52 (0.94%)	0.0004	0.0160	ko04730
Phototransduction - fly	58 (1.05%)	0.0005	0.0181	ko04745
Pancreatic secretion	100 (1.81%)	0.0005	0.0181	ko04972
Fanconi anemia pathway	43 (0.78%)	0.0012	0.0351	ko03460

E: Up-regulated DEGs between CK_6 and D_1G

KEGG pathway	DEGs genes with pathway annotation (4180)	*P* value	Q value	KO ID
Metabolic pathways	925 (22.13%)	0.0000	0.0000	ko01100
Chemical carcinogenesis	115 (2.75%)	0.0000	0.0000	ko05204
Pentose and glucuronate interconversions	104 (2.49%)	0.0000	0.0000	ko00040
Retinol metabolism	119 (2.85%)	0.0000	0.0000	ko00830
Metabolism of xenobiotics by cytochrome P450	106 (2.54%)	0.0000	0.0000	ko00980
Steroid hormone biosynthesis	82 (1.96%)	0.0000	0.0000	ko00140
Bile secretion	114 (2.73%)	0.0000	0.0000	ko04976
Ascorbate and aldarate metabolism	72 (1.72%)	0.0000	0.0000	ko00053
Drug metabolism - other enzymes	98 (2.34%)	0.0000	0.0000	ko00983
Drug metabolism - cytochrome P450	92 (2.2%)	0.0000	0.0000	ko00982
Biosynthesis of amino acids	97 (2.32%)	0.0000	0.0000	ko01230
Porphyrin and chlorophyll metabolism	64 (1.53%)	0.0000	0.0000	ko00860
Biosynthesis of unsaturated fatty acids	34 (0.81%)	0.0000	0.0000	ko01040
Carbon metabolism	120 (2.87%)	0.0000	0.0000	ko01200
Fatty acid metabolism	61 (1.46%)	0.0000	0.0000	ko01212
Selenocompound metabolism	31 (0.74%)	0.0000	0.0000	ko00450
Ether lipid metabolism	42 (1%)	0.0000	0.0000	ko00565
Glycine, serine and threonine metabolism	46 (1.1%)	0.0000	0.0000	ko00260

Peroxisome	96 (2.3%)	0.0000	0.0000	ko04146
Arachidonic acid metabolism	50 (1.2%)	0.0000	0.0000	ko00590
ABC transporters	51 (1.22%)	0.0000	0.0002	ko02010
Fructose and mannose metabolism	46 (1.1%)	0.0000	0.0002	ko00051
Folate biosynthesis	36 (0.86%)	0.0000	0.0002	ko00790
Ferroptosis	35 (0.84%)	0.0000	0.0002	ko04216
PPAR signaling pathway	61 (1.46%)	0.0000	0.0004	ko03320
Pyruvate metabolism	55 (1.32%)	0.0001	0.0009	ko00620
Salivary secretion	100 (2.39%)	0.0001	0.0010	ko04970
Lysosome	144 (3.44%)	0.0001	0.0012	ko04142
Riboflavin metabolism	22 (0.53%)	0.0001	0.0012	ko00740
Glycerolipid metabolism	60 (1.44%)	0.0001	0.0016	ko00561
Mineral absorption	44 (1.05%)	0.0002	0.0019	ko04978
Thyroid hormone signaling pathway	156 (3.73%)	0.0002	0.0020	ko04919
Fatty acid elongation	26 (0.62%)	0.0002	0.0022	ko00062
Endocrine and other factor-regulated calcium reabsorption	52 (1.24%)	0.0004	0.0038	ko04961
Protein digestion and absorption	95 (2.27%)	0.0006	0.0056	ko04974
Adipocytokine signaling pathway	57 (1.36%)	0.0009	0.0080	ko04920
Glutathione metabolism	53 (1.27%)	0.0009	0.0083	ko00480
Proximal tubule bicarbonate reclamation	36 (0.86%)	0.0010	0.0087	ko04964
Sulfur metabolism	21 (0.5%)	0.0013	0.0105	ko00920
Cardiac muscle contraction	42 (1%)	0.0013	0.0105	ko04260
Linoleic acid metabolism	20 (0.48%)	0.0013	0.0105	ko00591
One carbon pool by folate	17 (0.41%)	0.0013	0.0106	ko00670
Carbohydrate digestion and absorption	35 (0.84%)	0.0021	0.0159	ko04973
Insulin secretion	75 (1.79%)	0.0022	0.0167	ko04911
Glucagon signaling pathway	83 (1.99%)	0.0027	0.0195	ko04922
Cysteine and methionine metabolism	47 (1.12%)	0.0034	0.0243	ko00270
Thyroid hormone synthesis	58 (1.39%)	0.0038	0.0266	ko04918
Antifolate resistance	29 (0.69%)	0.0041	0.0283	ko01523
alpha-Linolenic acid metabolism	18 (0.43%)	0.0046	0.0311	ko00592
Pancreatic secretion	74 (1.77%)	0.0056	0.0360	ko04972
Arginine biosynthesis	20 (0.48%)	0.0056	0.0360	ko00220
Vitamin digestion and absorption	22 (0.53%)	0.0069	0.0440	ko04977

F: Down-regulated DEGs between CK_6 and D_1G

KEGGpathway	DEGs genes with pathway annotation (2698)	*P* value	Q value	KO ID
Hippo signaling pathway	72 (2.67%)	0.0000	0.0038	ko04390
Focal adhesion	112 (4.15%)	0.0002	0.0244	ko04510
Platelet activation	97 (3.6%)	0.0002	0.0244	ko04611
Fructose and mannose metabolism	30 (1.11%)	0.0004	0.0329	ko00051
Long-term potentiation	45 (1.67%)	0.0006	0.0384	ko04720

G: Up-regulated DEGs between CK_6 and D_2G

KEGG pathway	DEGs genes with pathway annotation (3515)	*P* value	Q value	KO ID
Ribosome	127 (3.61%)	0.0000	0.0000	ko03010
2-Oxocarboxylic acid metabolism	32 (0.91%)	0.0000	0.0002	ko01210
Chemical carcinogenesis	67 (1.91%)	0.0000	0.0014	ko05204
Retinol metabolism	74 (2.11%)	0.0000	0.0014	ko00830
Spliceosome	153 (4.35%)	0.0000	0.0014	ko03040
Pentose and glucuronate interconversions	59 (1.68%)	0.0000	0.0022	ko00040
Metabolism of xenobiotics by cytochrome P450	62 (1.76%)	0.0001	0.0055	ko00980
PPAR signaling pathway	50 (1.42%)	0.0003	0.0123	ko03320
Endocytosis	123 (3.5%)	0.0003	0.0123	ko04144
Glycerolipid metabolism	51 (1.45%)	0.0004	0.0124	ko00561
Tryptophan metabolism	33 (0.94%)	0.0005	0.0146	ko00380
Phenylalanine metabolism	20 (0.57%)	0.0007	0.0183	ko00360
Porphyrin and chlorophyll metabolism	40 (1.14%)	0.0007	0.0183	ko00860
Ascorbate and aldarate metabolism	43 (1.22%)	0.0008	0.0194	ko00053
Drug metabolism - other enzymes	62 (1.76%)	0.0009	0.0204	ko00983
Biosynthesis of amino acids	62 (1.76%)	0.0010	0.0210	ko01230
Ubiquinone and other terpenoid-quinone biosynthesis	16 (0.46%)	0.0011	0.0219	ko00130
Valine, leucine and isoleucine biosynthesis	8 (0.23%)	0.0015	0.0265	ko00290
Steroid hormone biosynthesis	47 (1.34%)	0.0015	0.0265	ko00140
Longevity regulating pathway - worm	57 (1.62%)	0.0017	0.0273	ko04212
Ether lipid metabolism	28 (0.8%)	0.0022	0.0338	ko00565
Fat digestion and absorption	23 (0.65%)	0.0039	0.0556	ko04975

H: Down-regulated DEGs between CK_6 and D_2G

KEGG pathway	DEGs genes with pathway annotation (3654)	*P* value	Q value	KO ID
Neuroactive ligand-receptor interaction	132 (3.61%)	0.0000	0.0000	ko04080
Phototransduction - fly	50 (1.37%)	0.0000	0.0003	ko04745
Gastric acid secretion	77 (2.11%)	0.0000	0.0003	ko04971
Insulin secretion	79 (2.16%)	0.0000	0.0004	ko04911
Glucagon signaling pathway	86 (2.35%)	0.0000	0.0005	ko04922
Pancreatic secretion	78 (2.13%)	0.0000	0.0009	ko04972
Proximal tubule bicarbonate reclamation	37 (1.01%)	0.0000	0.0016	ko04964
cAMP signaling pathway	136 (3.72%)	0.0000	0.0018	ko04024
Cysteine and methionine metabolism	49 (1.34%)	0.0001	0.0019	ko00270
Calcium signaling pathway	97 (2.65%)	0.0001	0.0019	ko04020
cGMP-PKG signaling pathway	116 (3.17%)	0.0001	0.0030	ko04022
Aldosterone synthesis and secretion	70 (1.92%)	0.0001	0.0035	ko04925
Oxytocin signaling pathway	101 (2.76%)	0.0003	0.0075	ko04921
D-Glutamine and D-glutamate metabolism	8 (0.22%)	0.0005	0.0111	ko00471
Arginine biosynthesis	20 (0.55%)	0.0012	0.0250	ko00220

Endocrine and other factor-regulated calcium reabsorption	45 (1.23%)	0.0012	0.0250	ko04961
Renin secretion	56 (1.53%)	0.0014	0.0250	ko04924
Pyruvate metabolism	45 (1.23%)	0.0014	0.0250	ko00620
Serotonergic synapse	83 (2.27%)	0.0015	0.0262	ko04726
Long-term potentiation	55 (1.51%)	0.0017	0.0267	ko04720
Melanogenesis	61 (1.67%)	0.0018	0.0275	ko04916
Thyroid hormone signaling pathway	132 (3.61%)	0.0020	0.0301	ko04919
Salivary secretion	82 (2.24%)	0.0024	0.0333	ko04970
GnRH signaling pathway	59 (1.61%)	0.0034	0.0455	ko04912
ErbB signaling pathway	42 (1.15%)	0.0036	0.0469	ko04012
Nitrogen metabolism	16 (0.44%)	0.0039	0.0484	ko00910
Glutamatergic synapse	56 (1.53%)	0.0041	0.0487	ko04724
